# A New Ophthalmosaurid (Ichthyosauria) from Svalbard, Norway, and Evolution of the Ichthyopterygian Pelvic Girdle

**DOI:** 10.1371/journal.pone.0169971

**Published:** 2017-01-25

**Authors:** Lene Liebe Delsett, Aubrey J. Roberts, Patrick S. Druckenmiller, Jørn H. Hurum

**Affiliations:** 1 Natural History Museum, University of Oslo, Oslo, Norway; 2 The National Oceanography Centre, Department of Ocean and Earth Science, University of Southampton, Southampton, Hampshire, United Kingdom; 3 University of Alaska Museum, Fairbanks, Alaska; 4 Department of Geoscience, University of Alaska Fairbanks, Fairbanks, Alaska; Institute of Botany, CHINA

## Abstract

In spite of a fossil record spanning over 150 million years, pelvic girdle evolution in Ichthyopterygia is poorly known. Here, we examine pelvic girdle size relationships using quantitative methods and new ophthalmosaurid material from the Slottsmøya Member Lagerstätte of Svalbard, Norway. One of these new specimens, which preserves the most complete ichthyosaur pelvic girdle from the Cretaceous, is described herein as a new taxon, *Keilhauia nui* gen. et sp. nov. It represents the most complete Berriasian ichthyosaur known and the youngest yet described from the Slottsmøya Member. It is diagnosed on the basis of two autapomorphies from the pelvic girdle, including an ilium that is anteroposteriorly expanded at its dorsal end and an ischiopubis that is shorter or subequal in length to the femur, as well as a unique character combination. The Slottsmøya Member Lagerstätte ichthyosaurs are significant in that they represent a diverse assemblage of ophthalmosaurids that existed immediately preceding and across the Jurassic–Cretaceous boundary. They also exhibit considerable variation in pelvic girdle morphology, and expand the known range in size variation of pelvic girdle elements in the clade.

## Introduction

Ichthyopterygia is a diverse clade of secondarily aquatic reptiles that lived from the Early Triassic (Olenekian) to the early Late Cretaceous (Cenomanian) [1, 2]. The clade had, by the Late Jurassic, developed into derived ichthyosaurs with a spindle-shaped body, large eyes and a small hind fin. The ichthyosaur body shape and its resemblance to odontocete cetaceans (especially delphinids and phocenids), is often used as a textbook example of convergent evolution among secondarily aquatic vertebrates.

One of the most important trends in ichthyosaur evolution relates to swimming modes and becoming high-speed pursuit predators. This evolutionary pathway is clearly seen in the posterior appendicular skeleton. Among terrestrial tetrapods, the pelvic girdle is necessary to articulate the vertebral column with the hind limbs in order to support the weight of the body and facilitate locomotion. Typically, the hind limbs are longer than the forelimbs and more commonly used for locomotion [3]. In contrast, it is assumed that derived ichthyosaurs did not use their limbs for locomotion [1, 4, 5]; particularly the hind fins, resulting in a reduction in pelvic girdle size and fusion of the ischium and pubis. Although ichthyosaur hind fins were reduced in size compared to the forefins, there is currently no evidence to suggest complete loss of the hind fin, as occurred among cetaceans [5–7].

An unanswered question is how the body shape evolution through the Jurassic and the Cretaceous affected the relative size and morphology of the pelvic girdle and hind fin. Pelvic girdles are likely important for inferring phylogenetic relationships and have been suggested to show a larger morphological variation than the pectoral girdle [8], but the degree of individual and intraspecific variation is currently not well understood. In spite of their potential importance, ichthyosaur pelvic girdles are relatively understudied (see overviews in dal Sasso and Pinna [9], Motani [10], Maisch and Matzke [11], McGowan and Motani [6]). Traditional descriptions and character matrices used in recent papers are skeletally biased towards the cranium and forefin; in comparison the pelvic girdle has received less attention as a source of useful taxonomic and phylogenetic data (but see new characters in [12]) [6, 13]. Compounding the problem are taphonomic biases, given the relatively small size of the pelvic girdle and hind fin and loose nature of their articulation with other pelvic elements and the vertebral column. Thus, these elements are more easily disarticulated from a rotting carcass during floating or disconnected when landing on the sea bottom [6, 14]. A similar bias exists for the reduced pelvic girdle (innominate) in many cetaceans [15]. Innominates are poorly known in many extinct cetacean species, particularly among fully aquatic taxa, and are virtually unknown in the gap between basilosaurids (late middle Eocene–late Oligocene) and extant species [16].

Many small, fully articulated ichthyosaur specimens from Triassic and Early to Middle Jurassic Lagerstätten have complete pelvic girdles, but this is not the case for the majority of late Middle Jurassic to Cretaceous ophthalmosaurids [8, 17] (S1 Table), in spite of the description of numerous new specimens and taxa in the last decade [18–25]. For five ophthalmosaurid genera, only the ischiopubis is known from the pelvic girdle. The only pelvic girdle material (an ischiopubis) of specimens referred to *Platypterygius* was recently described in *P*. *australis* [26].

**Fig 1:**
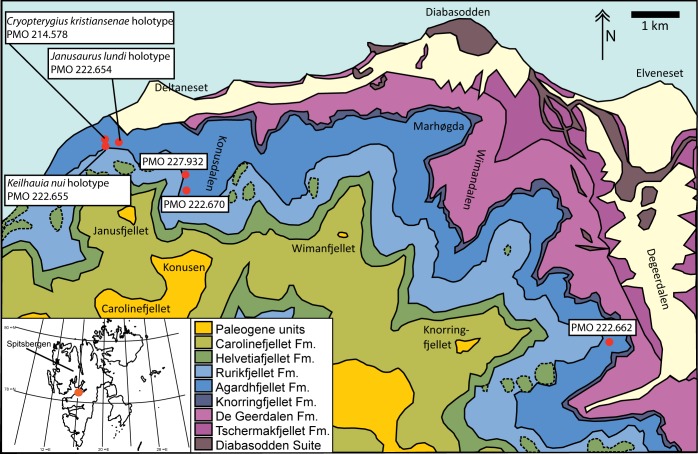
Map showing six ichthyosaur specimens from the Slottsmøya Member Lagerstätte discussed in the text (red dots). Overview map (left corner) shows the Svalbard archipelago and island of Spitsbergen; orange dot corresponds to excavation area. Adapted from Hurum et al. (2012).

**Fig 2:**
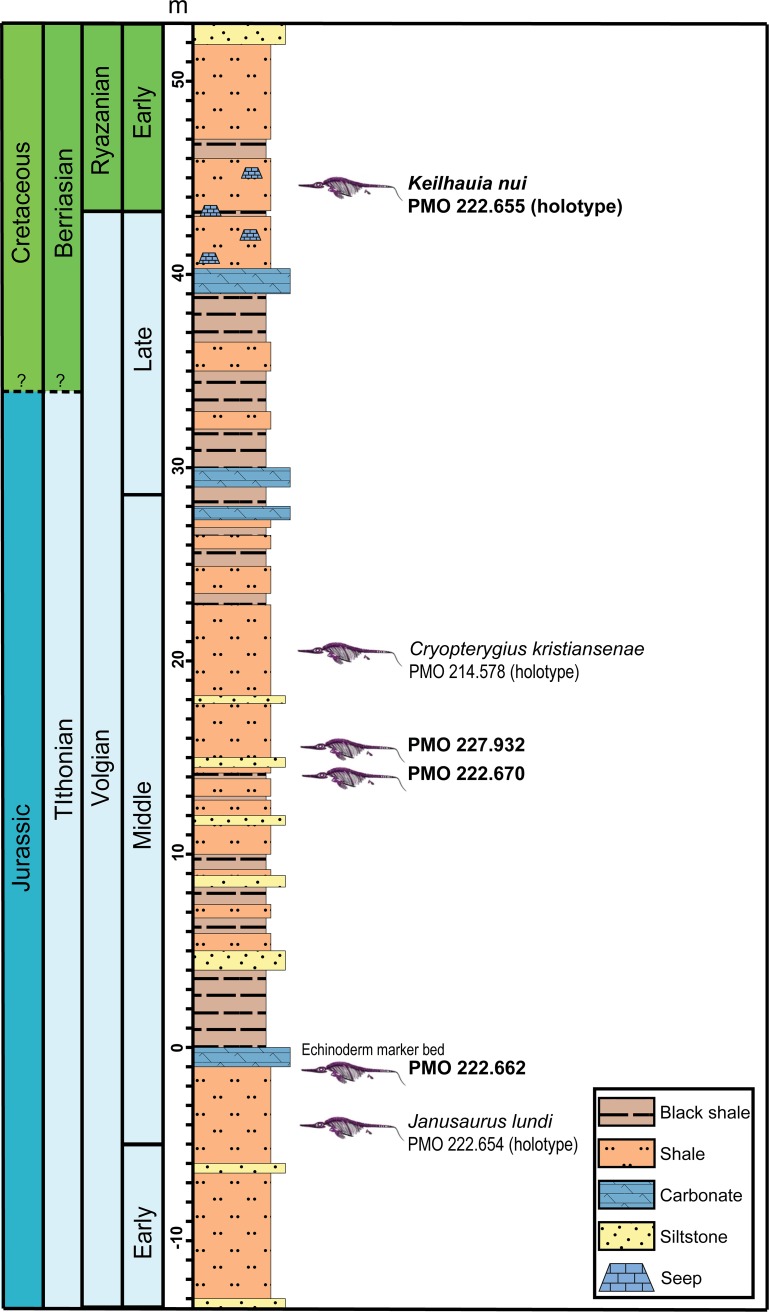
Stratigraphic section for the Slottsmøya Member Lagerstätte showing the six ichthyosaur specimens with pelvic girdles discussed in the text (newly described material shown in bold). Modified from Delsett et al. (2016).

Between 2004 and 2012 the Spitsbergen Mesozoic Research Group excavated the remains of 29 ichthyosaur specimens from the Late Jurassic–Early Cretaceous Slottsmøya Member Lagerstätte [27, 28] (Fig 1). Two previously described ichthyosaurs from the unit have contributed significantly to our knowledge of ichthyosaurian pelvic girdles (Fig 2). The holotype of *Cryopterygius kristiansenae* Druckenmiller, Hurum, Knutsen and Nakrem 2012 preserves the left ischiopubis and ilium together with the majority of an articulated hind fin. The holotype of *Janusaurus lundi* Roberts, Druckenmiller, Sætre and Hurum 2014 preserves the left ischiopubis, both ilia and two disarticulated hind fins [25, 29].

In addition to these previously described specimens, four other Slottsmøya Member specimens have pelvic material, and are described herein. One specimen is Berriasian in age, and consists of a partial skeleton including the most complete pelvic girdle from the Cretaceous known to date. Ichthyosaurs are very poorly known from the Berriasian-Barremian interval [19, 30] with only fragmentary Berriasian material known from a few specimens in England [19, 31, 32], Argentina [17, 33], and possibly from Russia [34] and Chile [35]. The second partial specimen described here is from the Tithonian and is significant because it preserves a complete pelvic girdle with both femora, previously only known from one Late Jurassic specimen except *Ophthalmosaurus icenicus* and the holotype of *Janusaurus lundi* [29], from which it differs substantially. Finally, two additional Tithonian specimens consist of pelvic girdle and hind fin material.

The aims of this paper are to: 1) describe four new ichthyosaur specimens from the Slottsmøya Member Lagerstätte; 2) conduct a phylogenetic analysis to determine the position of the most complete specimen, PMO 222.655; 3) use the new material to address size relationships between pelvic girdle elements and femora in Jurassic and Cretaceous taxa and 4) provide an overview of the shape of pelvic girdle elements in ichthyosaurs from the Triassic to their extinction in the Cretaceous and compare this evolution to that of the cetacean innominate.

### Institutional abbreviations

SNSB-BSPG, Bayerische Staatssammlung für Paläontologie und Geologie Munich; CAMSM, Sedgwick Museum of Earth Sciences; EP, Paleontological Museum of Undory, Ul’yanovsk Region, Russia; GLAHM, The Hunterian Museum, University of Glasgow; LEIUG, University of Leicester; NHMUK, Natural History Museum, UK; OUMNH, Oxford University Museum; PMO, Natural History Museum, Oslo, paleontological collections; TMP, Royal Tyrrell Museum of Palaeontology, Drumheller, Alberta, Canada.

## Geological Setting

The ichthyosaur specimens described in this study were recovered from the Late Jurassic–Early Cretaceous Slottsmøya Member Lagerstätte on Spitsbergen (Fig 2), the largest island in the Svalbard archipelago (74°–81° North, 10°–35° East)(Fig 1). The Slottsmøya Member is the uppermost member in the Agardhfjellet Formation, a 90–350 metres thick unit in the Janusfjellet Subgroup, Adventdalen Group [36]. The Adventdalen Group spans the Middle Jurassic to Lower Cretaceous. During deposition of the Slottsmøya Member, Svalbard was situated at 63–66° North [37] and largely covered by an epicontinental sea situated in the Boreal Basin [38, 39].

The Slottsmøya Member is a 70–100 m thick, upwards-coarsening unit made up primarily of dark-grey to black shales and paper shales [38, 40]. Silty and sandy beds also occur, as well as some carbonates. The member was deposited on a slightly dysoxic open marine shelf with periodic oxygenation of the sea bottom [41]. The siltstones and sandstones are interpreted as the result of storms or turbiditic currents [40]. A continuous yellow siltstone bed rich in echinoderm fossils is used as a marker unit and set as 0 m in the section (“echinoderm marker bed”) [27]; the position of the specimens described here is given relative to this bed (Fig 2). The distribution of the marine reptiles, invertebrates and total organic content fluctuations in the section is described in detail in Delsett et al. [28].

Based on ammonite and foraminiferal biostratigraphy, the Slottsmøya Member spans the Jurassic–Cretaceous boundary and is upper Tithonian to upper Berriasian in age [42–44]. A regional chronostratigraphic framework for the latest Jurassic and earliest Cretaceous in the Boreal region uses the terms Volgian and Ryazanian to broadly correspond to the Tithonian and Berriasian in wide use elsewhere (Fig 2). Currently, it is unclear whether the Volgian and Ryzanian border corresponds precisely to the Tithonian and Berriasian boundary, i.e. between the Jurassic and the Cretaceous (e.g. [45, 46]). Because Tithonian and Berriasian are more commonly used, these names will be used in this paper.

## Material and Methods

### Material

Four new ichthyosaur specimens are described here: PMO 222.655 (collected 2010), PMO 222.670 (collected 2011), PMO 222.662 (collected 2007) and PMO 227.932 (collected 2011), which are widely distributed throughout the Slottsmøya Member (Fig 2). The stratigraphic position of each specimen is well constrained and was determined using a total station [27, 40]. Marine reptile specimens from the Slottsmøya Member Lagerstätte display a range of preservation modes from full articulation to disarticulation, and varying preservation of individual elements. Many elements are severely broken and eroded, but compaction and compression is less pronounced than what would usually be expected in these types of sediments. The three-dimensionality is likely a result of early diagenetic barite precipitation in the pore spaces of the bones [28]. The specimens were transported from the field in plaster jackets and mechanically prepared in the lab at the Natural History Museum, University of Oslo.

### Calculation of relative lengths of elements

One of the aims of this study is to understand the size relationships between pelvic girdle and limb elements of ichthyosaurs from the Early Jurassic to the Cretaceous. To do this, the length of different elements (humerus, ilium, ischiopubis and femur) were measured and calculated as simple ratios to investigate whether there are any clear evolutionary trends or unique pelvic girdle architecture among the different species. The ratios were also plotted against geological ages for each taxon to test for correlations.

Measurements were taken from Jurassic and Cretaceous specimens that preserve at least two of the following lengths: humerus, ilium, ischiopubis and femur. In specimens with two humeri or femora, the mean proximodistal length was used (measurement details are provided in S1 Text). It should be noted that the time range for comparisons of ilial and femoral length to ischiopubic length is shorter than when compared to other elements, due to the change from tripartite (the pelvic girdle consists of ilium, ischium and pubis) to bipartite (ilium and ischiopubis) pelvic girdles. More complete or near-complete specimens with pelvic girdles are known for Early–Middle Jurassic taxa than from late Middle Jurassic to Cretaceous ophthalmosaurids, and *Stenopterygius* is unique for being known from numerous specimens. Both of these factors skew the dataset to some extent, and a Spearman rs correlation test is used. From Jurassic and Cretaceous taxa with known pelvic girdles (S1 Table), *Excalibosaurus* is the only genus not represented by any specimen in the calculations because we have either not personally examined this material or the necessary measurements are available in the published literature. Triassic taxa are outside the scope of this paper. For geological age a median age was used based on which stages the species is known to occur (see S2 Table for references).

### Statistical methods

A Spearman rs correlation test was conducted in PAST 3 [47] for the relationship between geological age and the following length ratios; femur: humerus; ilium: femur; ischiopubis: ilium and ischiopubis: femur. The specimens were grouped into four categories; Early/Middle Jurassic specimens with a tripartite pelvis, Early/Middle Jurassic juveniles with a bipartite pelvis (only *Stenopterygius* specimens), Early/Middle Jurassic adults with a bipartite pelvis, Late Jurassic specimens and Cretaceous specimens.

### Pelvic girdle evolution in a phylogenetic framework

To facilitate discussion about the evolution of pelvic girdle shape, illustrations of known pelvic girdles were prepared and plotted onto a recent phylogeny of Ichthyopterygia using the topology of Ji et al. [48] for non-ophthalmosaurids. Because an additional aim of this study is to establish the phylogenetic position of PMO 222.655 using a larger taxon sampling of derived ichthyosaurs than that presented in Ji et al. [48], a new phylogenetic analysis of Ophthalmosauridae was conducted using the matrix of Roberts et al. [29] (S2 Text and S3 Text for character list and data matrix). The new matrix (22 OTUs and 56 characters) was assembled and analyzed in TNT V1.1 and includes PMO 222.655; however the other new material described herein was excluded due to its fragmentary nature. The scores for *Undorosaurus gorodischensis* Efimov 1999 are based on personal observation (LLD at EP) and Arkhangelsky and Zverkov [49]. The matrix of Fischer et al. [50] was not used because only two of the novel post-cranial characters would be possible to score for PMO 222.655. The character matrix for ichthyosaurs is still very volatile, and several different sets are in current use (e.g. [12]). In addition, the slightly smaller data matrix of Roberts et al. [29] permits the use of implicit numeration as a search algorithm. All characters were unweighted and unordered. The analysis was performed in TNT V1.1 [51] and run using the implicit enumeration algorithm with the outgroup taxon specified as *Temnodontosaurus*. Bremer support was calculated using the bremer function in TNT. CI and RI statistics were found using the stats.run script for TNT.

### Nomenclatural acts

The electronic edition of this article conforms to the requirements of the amended International Code of Zoological Nomenclature, and hence the new names contained herein are available under that Code from the electronic edition of this article. This published work and the nomenclatural acts it contains have been registered in ZooBank, the online registration system for the ICZN. The ZooBank LSIDs (Life Science Identifiers) can be resolved and the associated information viewed through any standard web browser by appending the LSID to the prefix “http://zoobank.org/”. The LSID for this publication is: urn:lsid:zoobank.org:pub:8E049808-F51A-42C5-916B-D5AE05669596.

The electronic edition of this work was published in a journal with an ISSN, and has been archived and is available from the following digital repositories: PubMed Central, LOCKSS and CRIStin (University of Oslo Library).

### Systematic Paleontology

Ichthyosauria de Blainville 1835

Neoichthyosauria Sander 2000

Thunnosauria Motani 1999

Ophthalmosauridae Baur 1887

*Keilhauia* gen. nov.

urn:lsid:zoobank.org:act:2D45A81F-D4FB-4BA3-8F61-B9BB775389F0

*Keilhauia nui* sp. nov.

urn:lsid:zoobank.org:act:A48F4CB9-A930-4DB3-A737-004EBC772537

(Figs 3–9, Table 1)

**Fig 3:**
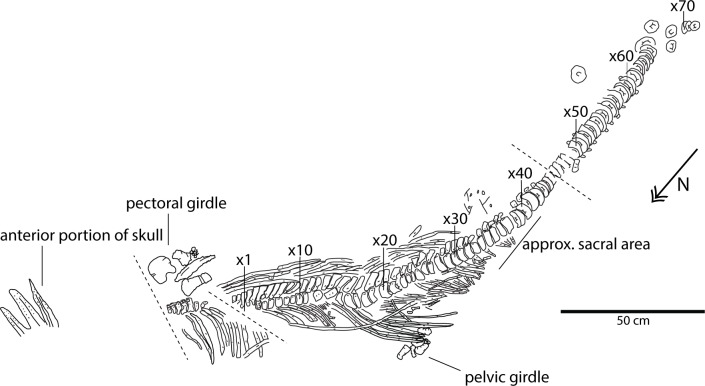
Skeletal map of *Keilhauia nui* (PMO 222.655) viewed from the side stratigraphically down, i.e. the prepared side. Vertebrae numbers (“x#”) indicate position relative to the anterior end of the preserved skeleton and do not correspond to their actual position in the column. Dashed lines show three faults. Scale bar equals 50 cm. Modified from Delsett et al. 2016.

**Fig 4:**
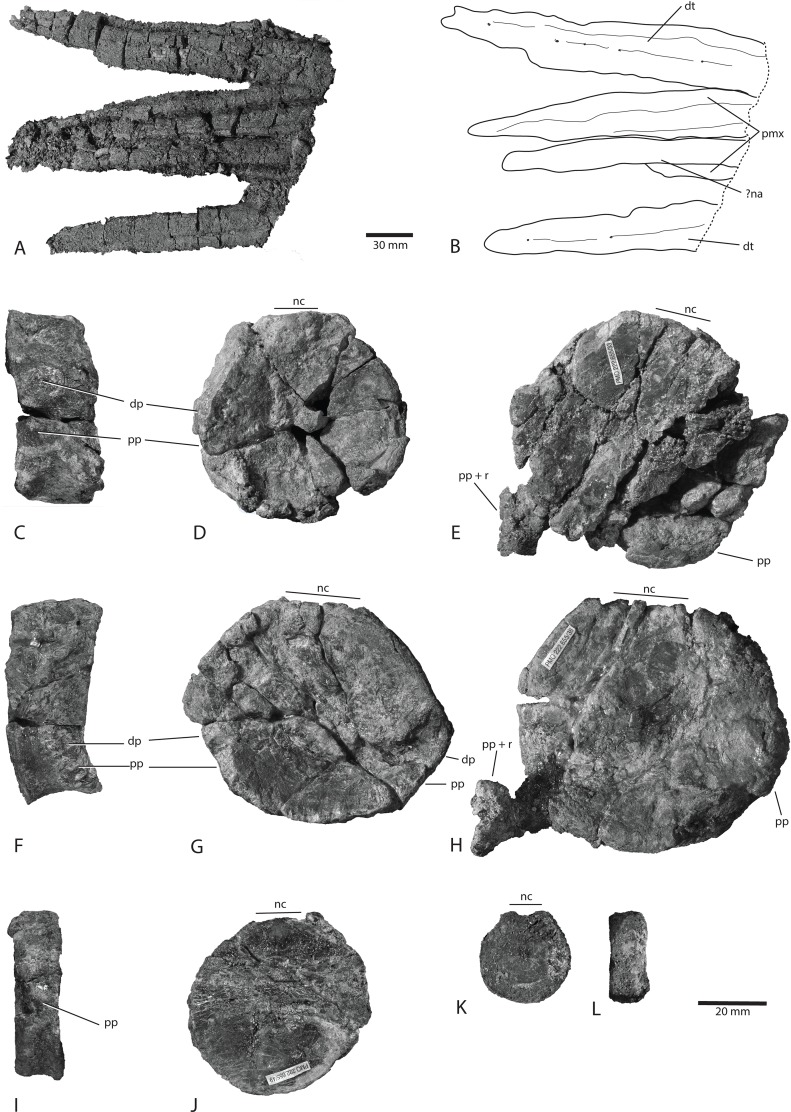
Rostrum and vertebrae of *Keilhauia nui* (PMO 222.655). Rostrum in A and B; dentaries in lateral view and premaxillae in ventral view. Scale bar equals 30 mm. Vertebra x18 (anterior dorsal) in C left lateral and D posterior views. Vertebra x39 (possible sacral) in E anterior view. Vertebra x29 (posterior dorsal) in F right lateral and G anterior views. Vertebra x54 (anterior caudal) in H anterior view. Vertebra x64 (caudal) in I lateral and J? anterior views. Vertebra x72 (fluke) in K anterior or posterior and L lateral views. Vertebrae numbers do not correspond to actual position in the vertebral column. Scale bar for C-L equals 20 mm. Abbreviations: **dp** diapophyses, **dt** dentary, **na** nasal, **nc** neural canal, **pmx** premaxilla, **pp** parapophyses, **r** rib.

**Fig 5:**
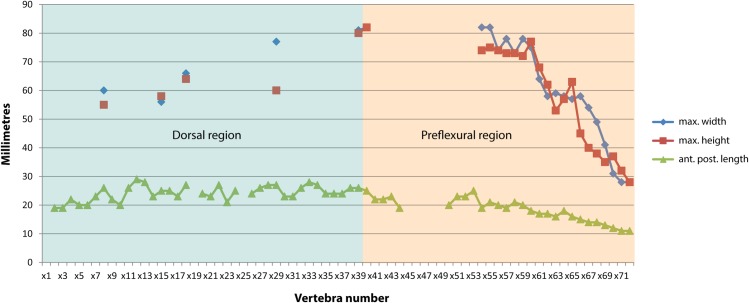
Vertebral dimensions of *Keilhauia nui* (PMO 222.655). Vertebral numbers do not correspond to the actual position in the column, but to those used in the text. Blue area: dorsal region, orange area: preflexural area.

**Fig 6:**
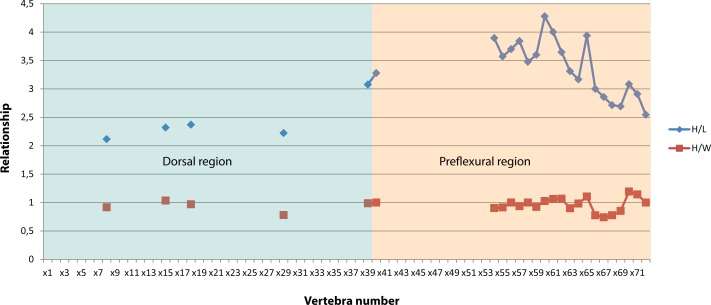
Ratios of vertebral dimensions of *Keilhauia nui* (PMO 222.655) showing the relationship between height to length and height to width. Vertebral numbers do not correspond to the actual position in the column, but to those used in the text. Blue area: dorsal region, orange area: preflexural area.

**Fig 7:**
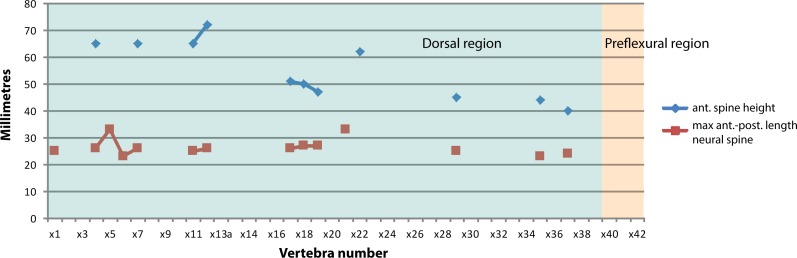
Neural spine dimensions of *Keilhauia nui* (PMO 222.655) showing height and maximum anterior-posterior length. Vertebrae numbers do not correspond to the actual position in the column, but to those used in the text. Blue area: dorsal region, orange area: preflexural area.

**Fig 8:**
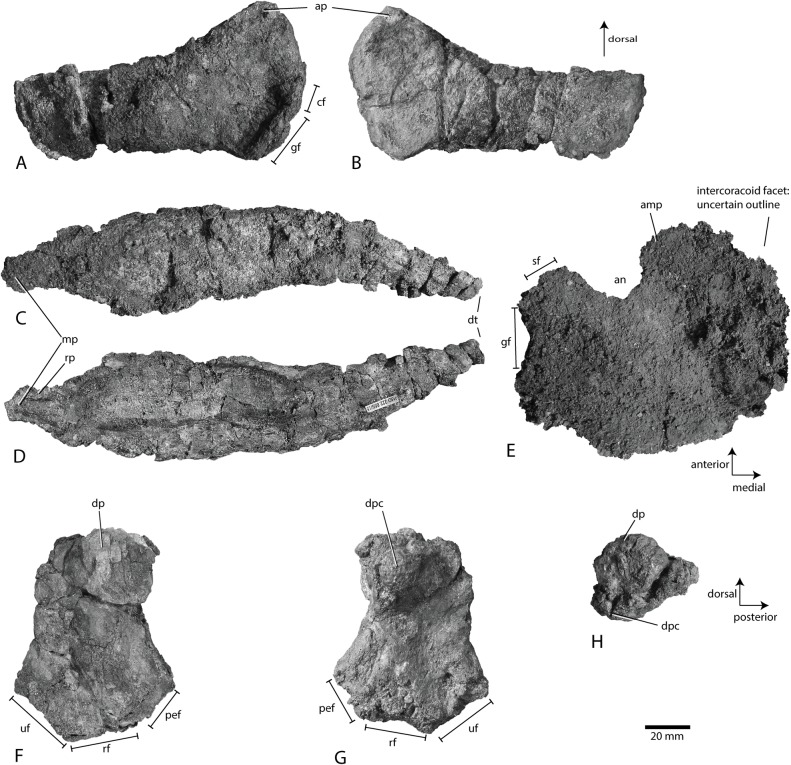
Right pectoral girdle and humerus of *Keilhauia nui* PMO 222.655. Scapula in A lateral and B medial views. Clavicle in C dorsal and D ventral views. Coracoid in E ventral view. Humerus in F dorsal, G ventral and H proximal views. Scale bar equals 20 mm. Abbreviations: **amp** anteromedial process, **an** anterior notch**, ap** acromion process, **cf** coracoid facet **dp** dorsal process, **dpc** deltopectoral crest, **dt** distal tip, **gf** glenoid facet, **mp** medial projection, **pef** preaxial accessory element facet, **rf** radial facet, **rp** rim on posterior side of medial projection, **sf** scapular facet, **uf** ulnar facet.

**Fig 9:**
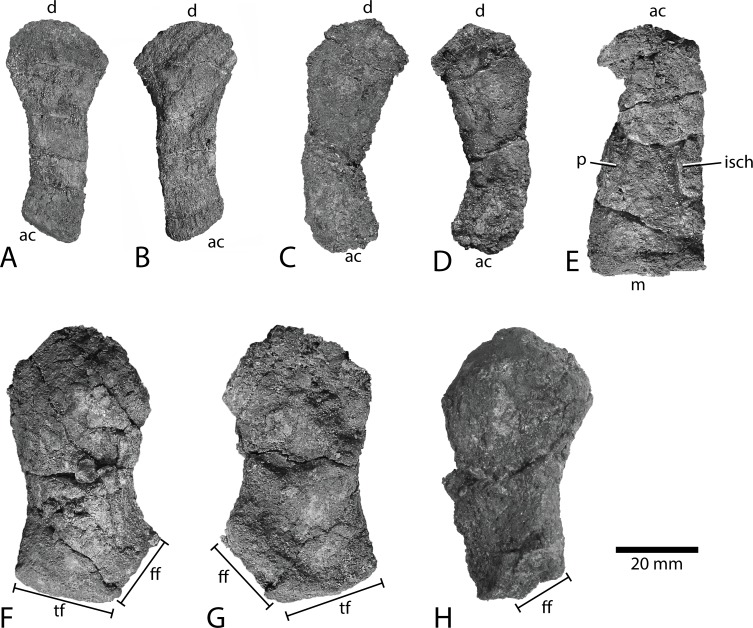
Pelvic girdle and femur of *Keilhauia nui* (PMO 222.655). Ilium in A and B lateral and medial (?) views. Posterior is to the left in A. The other ilium in C and D lateral (?) and medial (?) views. Posterior is to the right in C. Ischiopubis in E lateral or medial view. The better-preserved femur in F and G dorsal (?) and ventral (?) views. The other femur from the best preserved side in H dorsal or ventral view. Scale bar equals 20 mm. Abbreviations: **ac** acetabular end, **d** dorsal end, **ff** fibular facet, **isch** ischium, **m** medial end, **p** pubis, **tf** tibial facet.

**Table 1:** Selected measurements of PMO 222.655, holotype specimen of *Keilhauia nui*. Lengths given in millimetres.

**Right humerus PMO 222.655/1 (Fig 8F–8H)**	
Maximum proximodistal length	86
Minimum anteroposterior width, midshaft	44
Maximum anteroposterior width, proximal end	49
Maximum dorsoventral height, proximal end	40
Maximum anteroposterior width, distal end	67
Maximum dorsoventral height, distal end	25
Anteroposterior length of radial facet	30
Anteroposterior length of ulnar facet	26
Anteroposterior length of preaxial element facet	20
Maximum dorsoventral width of radial facet	17
Maximum dorsoventral width of ulnar facet	7
Maximum dorsoventral width of facet for preaxial acc. element	11
**Right scapula PMO 222.655/2 (Fig 8A and 8B)**	
Maximum proximodistal length	123
Maximum anteroposterior width, proximal blade	67
Minimum anteroposterior width, middle of blade	36
Maximum anteroposterior width, distal blade	34
Dorsoventral length of coracoid facet	17
Dorsoventral length of glenoid facet	24
**Coracoid PMO 222.655/17 (Fig 8E)**	
Maximum mediolateral width	110 (estimate)
Maximum anteroposterior length	118
Anteroposterior length of glenoid facet	62
Anteroposterior length of scapular facet	28
**Clavicle PMO 222.655/15 (Fig 8C and 8D)**	
Maximum width	45
Maximum length	204
**Ilium PMO 222.655/60 (Fig 9A and 9B)**	
Maximum dorsal-acetabular length	54
Maximum anteroposterior width, acetabular end	25
Maximum height (thickness), acetabular end	2
Maximum anteroposterior width, dorsal end	16
**Ilium PMO 222.655/61 (Fig 9C and 9D)**	
Maximum dorsal-acetabular length	55
Maximum anteroposterior width, acetabular end	24
Maximum height (thickness), acetabular end	3
Maximum anteroposterior width, dorsal end	16
**Ischiopubis PMO 222.655/62 (Fig 9E)**	
Maximum proximodistal length	59
Maximum anteroposterior width, proximal end	20
Maximum anteroposterior width, distal end	27
**Left femur PMO 222.655/58 (Fig 9F and 9G)**	
Maximum proximodistal length	64
Maximum anteroposterior width, proximal end	33
Maximum height, proximal end	14
Maximum anteroposterior width, distal end	33
Minimun anteroposterior width of shaft	25
Anteroposterior length of tibial facet	13
Anteroposterior length of fibular facet	14
**Right femur PMO 222.655/59 (Fig 9H)**	
Maximum proximodistal length (partly eroded)	60
Maximum anteroposterior width, proximal end	36
Maximum height, proximal end	16

**Holotype and only specimen:** PMO 222.655, an articulated, partial skeleton consisting of an incomplete rostrum, the dorsal and preflexural vertebrae, the right pectoral girdle and forefin, most of the pelvic girdle and both femora.

**Etymology:** Genus name in honor of Baltazar Mathias Keilhau (1797–1858), the first Norwegian geologist to do fieldwork in the Arctic. He was part of an expedition to Svalbard (Spitsbergen) in 1827. His collection is housed at the Natural History Museum in Oslo, Norway, where PMO 222.655 is also housed. Species name in honor of Natur og Ungdom (Young Friends of the Earth Norway) working to protect the Arctic environment, who celebrate their 50 year anniversary in 2017.

**Holotype locality:** Island of Spitsbergen, north side of Janusfjellet, approximately 13 km north of Longyearbyen, Svalbard, Norway. UTM WGS84 33X 0518847 8696044

**Holotype horizon and stage:** Slottsmøya Member, Agardhfjellet Formation, Janusfjellet Subgroup, early Berriasian, Early Cretaceous. 44.8 metres above the echinoderm marker bed.

#### Differential diagnosis

An ophthalmosaurid with the following autapomorphies: ilium anteroposteriorly expanded at the dorsal end; ischiopubis shorter or subequal in length to the femur (differs from all other Middle-Late Jurassic and Cretaceous ophthalmosaurids, but found in some specimens of *Stenopterygius* sp).

The species also possesses the following unique character combination: Posterior dorsal and anterior caudal centra less than 3.5 times as high as long (4 times or more in *Ophthalmosaurus icenicus* and *Arthropterygius chrisorum*)[52, 53]; glenoid contribution of the scapula larger than coracoid facet (smaller in *Caypullisaurus bonapartei* and *Sveltonectes insolitus*)[24, 54]; small acromion process of scapula (prominent in *Brachypterygius extremus*, *Acamptonectes densus*, *Platypterygius americanus*, *Caypullisaurus bonapartei* and *Sveltonectes insolitus*)[19, 24, 52, 54, 55]; anteromedial process of coracoid present (not present in *Caypullisaurus bonapartei* and *Platypterygius australis*)[26, 54]; absence of a strongly developed deltopectoral crest of the humerus (present in *Caypullisaurus bonapartei*, *Platypterygius americanus* and *Sveltonectes insolitus*)[24, 54, 55]; humerus with facet for preaxial accessory element anterior to radius (absent in *Brachypterygius extremus*, *Maiaspondylus lindoei*, *Aegirosaurus leptospondylus* and *Sveltonectes insolitus*)[24, 52, 56, 57]; posteriorly deflected ulnar facet (absent in *Brachypterygius extremus*, *Maiaspondylus lindoei*, *Caypullisaurus bonapartei*, *Platypterygius australis*, *Aegirosaurus leptospondylus*, *Sveltonectes insolitus* and *Cryopterygius kristiansenae*)[24–26, 52, 54, 56, 57]; lack of a contact between the humerus and intermedium (present in *Brachypterygius extremus*, *Maiaspondylus lindoei* and *Aegirosaurus leptospondylus*)[52, 56, 57]; complete fusion of the ischium and pubis lacking an obturator foramen (only proximal fusion in *Cryopterygius kristiansenae* and *Undorosaurus gorodischensis*, present with foramen in *Ophthalmosaurus icenicus*)[25, 52, 58]; medial margin of ischiopubis straight in dorsal and ventral view (shared with *Janusaurus lundi* only)[29]; femur with two distal facets (three in *Platypterygius americanus*, *P*. *australis*, *P*. *hercynicus* and *Paraophthalmosaurus saratoviensis*)[26, 55, 59, 60].

#### Preservation

PMO 222.655 is articulated and partially complete (Fig 3). The individual appears to have landed on the seafloor on its left lateral side. Skeletal preservation improves posteriorly. The only preserved portion of the skull are the tips of the rostral elements (length = 25 cm), including the anterior portions of the dentaries and premaxillae, and a smaller portion of the nasal (Fig 4). A few isolated partial teeth were found in association with fragments of the ribs and gastralia. The teeth most likely originate from the skull of PMO 222.655, and are probably not gut content, judging from their size. Almost the entire vertebral column is preserved in articulation. For the purposes of description, numbers of individual vertebrae (x1-x72) and neural arches (x1-x42) were given during preparation and indicate position relative to the anterior end of the preserved skeleton but do not correspond to their actual position in the column (Figs 5–7). Some vertebrae are probably missing in the cervical region. Anterior to the fault in the anterior dorsal region, the individual vertebrae are difficult to discern, and directly posterior to it, severely crushed. Vertebra x19 is not preserved, but its existence is indicated by a higher number of neural arches than vertebrae in this area. Most of the vertebrae preserve their neural arches. Anterior to the fault in the anterior dorsal region, seven vertebrae have parts of their neural arches. Posterior to this fault 43 neural arches are preserved, the most complete are those belonging to vertebrae x3-x6. Between the body and the preserved part of the tail is a compressed area with fragments of five vertebrae. The entire postflexural column is missing. Many ribs are preserved in articulation. Anterior to the fault in the anterior dorsal region, the ribs are in position but fractured. Posterior to this fault they are relatively well preserved, but almost none are complete. Fourty-one ribs are preserved in position on the left lateral side of the specimen and 21 on the right side. The left side of the pectoral girdle (Fig 8) is missing, as well as the left forefin and one of the ischiopubes. The pelvic girdle and hind fin (Figs 3 and 9) appear to have drifted or been moved anteriorly after death because the elements was found at the position of vertebra x20, whereas the sacral vertebra is interpreted to be vertebra x39 (see below).

#### Ontogeny

The proximal head of the humerus is convex (Fig 8H), which is traditionally considered a criterion for skeletal maturity [61]. The surface texture of the humerus shaft is partly eroded, but is relatively smooth and not “sand paper-like” as would be expected in a juvenile specimen [61]. The degree of ossification (when it is possible to observe) resembles mature finished bone [61]. The closure of the gaps between proximal fin elements could not be assessed because the forefin was not in articulation [61]. Criteria relating to the orbit and sclerotic aperture, the basicranium and the neural spines [62] are not applicable because these parts are not preserved or poorly preserved. Based on available evidence and the size of the animal, we infer that PMO 222.655 reached a late juvenile to adult ontogenetic stage.

#### Total body length

Estimated total body length is based on a combination of the length of the vertebral column and rostrum, an estimation of missing portions and relative position using the quarry map. The unpreserved part of the skull is assumed to represent two thirds of total skull length (50 cm) based on comparable skull dimensions of *Cryopterygius kristiansenae* (PMO 214.578) [25]. The preserved part of the vertebral column has a length of 260 cm. The missing cervical vertebrae are estimated to represent 10–20 cm, and the compressed caudal area might have extended the measureable body length up to 10 cm. These lengths depend on how many vertebrae are missing and how much the compression expanded the area anterior to the tail. We assume that the missing part is the fluke and that it was approximately 60–80 cm in length, based on the relative size of the fluke of *Ophthalmosaurus icenicus* [7]. This provides an estimated total body length of 3.8–4.3 metres.

#### Skull

The premaxillae and nasals are not preserved well enough to present any details; however, the rostrum of *Keilhauia nui* (PMO 222.655) (Fig 4A and 4B) becomes dorsoventrally taller compared to the equivalent point in the rostrum of *Aegirosaurus leptospondylus* Wagner 1853 [56], and is more similar to that of *Cryopterygius kristiansenae* [25] and *Caypullisaurus bonapartei* Fernández 1997 [17, 54]. Nutrient foramina are visible on the lateral sides of the dentaries in a shallow longitudinal groove reaching almost to the anterior tip of these elements. Fragments from approximately ten teeth are preserved. None of them are complete, and none preserve the enamel, so that length and possible ornamentation is not possible to describe. The crowns that are preserved are conical, with a narrow crown apex and a slight lingual curvature.

#### Vertebral column

Thirty-eight more or less well preserved vertebrae are preserved in the presacral region and 33 in the caudal (Figs 3 and 4C–4L). Additionally, poorly preserved remains of 5–7 vertebrae are located in the anteriormost area of the body, which are neither given numbers in the “x” series nor measured. Centrum number x39 (Figs 4E, 5 and 6) marks the approximate position of the sacral vertebra based on the observations that it possesses only one apophysis and thus might represent the transition from bicipital to unicipital ribs, and that rib length rapidly decreases from vertebra x28 (10 cm) to x39 (4 cm). However, the transition from bicipital to unicipital ribs could lie anterior to this because not all of the centra were removed from the matrix for closer examination. An exact presacral vertebral count is not possible to determine; however, based on the preserved centra, the presacral number must have been at least 43. This is more than in *Nannopterygius enthekiodon* Hulke 1871 (41 vertebrae [52]), *Platypterygius americanus* Nace 1939 (37 vertebrae [55]) and *Athabascasaurus bitumineus* Druckenmiller and Maxwell 2010 (42 vertebrae[22]). There is uncertainty regarding the number of presacral vertebrae in *Ophthalmosaurus icenicus* Seeley 1874, with estimates ranging from 38–43 [7, 52]. *Platypterygius australis* McCoy 1867 has 46 presacral vertebrae, [26], and *Cryopterygius kristiansenae*, *Aegirosaurus leptospondylus* and *Platypterygius platydactylus* Broili 1907 have more than 50 presacral vertebrae [25, 56, 63].

The height and width of the centra in the dorsal region increase posteriorly. In vertebra x18 (Fig 4C and 4D), the diapophysis and the parapophysis are situated midway between the dorsal and ventral edges on the lateral side, indicating that this is an anterior dorsal vertebra, and the apophyses on vertebra x29 (Fig 4F and 4G) are situated on the ventral half, typical of a posterior dorsal vertebra. The majority of the vertebrae from the dorsal region are circular in anterior or posterior view.

Generally, the caudal centra in the anterior part of the preflexural stock are the highest and widest in the entire column [7]. In *Keilhauia nui* (PMO 222.655), that is found at vertebra x40 (Figs 5 and 6). Similar to the dorsal vertebrae, the anterior caudal vertebrae are nearly circular in anterior or posterior view [7]. The largest height: length ratio is found in vertebra x59 (Fig 6) and decreases posterior to this. In the posterior part of the preflexural stock the height of the vertebrae decreases rapidly but the width decreases less rapidly. This results in dorsoventrally shortened vertebrae, which can be seen in centra x59-x71 (Fig 4I–4L). Vertebra x72 (Fig 4K and 4L) is higher than wide, an indication of the fluke base [7]. The centrum edges change from being sharp to broadly rounded shortly before the fluke [7], a feature that is observed from vertebra x69 and posteriorly. The apical centra were not preserved. Some small, rounded bones (diameter 10–15 mm) that were found in the region of vertebrae x50-x60 may be chevrons.

The neural spines are posteriorly inclined and of the same anteroposterior length throughout the axial skeleton, although in some cases the length expands slightly in the dorsalmost part (Fig 7). The anteroposterior length is similar to that of their corresponding vertebral centra. This is similar to *Cryopterygius kristiansenae* [25] but differs from *Ophthalmosaurus icenicus*, where the anteroposterior length increases posterior to vertebra 25 [52]. The mediolateral width of the neural spine is narrowest at the dorsal end (approximately 5 mm), and increases ventrally. The dorsal margins of the spines are straight and lack the dorsal notches as seen in *Cryopterygius kristiansenae* [25] or *Platypterygius australis* [64]. Whether the dorsal margins are rugose or have a longitudinal groove as in *Ophthalmosaurus icenicus* [52] for an extension of soft tissue is not possible to determine. The dorsal end of the neural spines in *Aegirosaurus leptospondylus* (SNSS-BSPG 1954 I 608) is smoother than in *Keilhauia nui* (PMO 222.655) and slightly convex. The neural spines are similar in height but the height drops by 30% at vertebra x20, and continues to decrease after that (Fig 7). In *Ophthalmosaurus icenicus* the neural spines decrease rapidly in height in the posterior trunk region. However, the maximum neural spine height is encountered approximately at vertebra 25 in *Ophthalmosaurus icenicus* [52], but this happens in the anterior dorsal section in *Keilhauia nui* (PMO 222.655). In the anteriormost preserved vertebrae in *Keilhauia nui*, the neural spine height is larger than the corresponding centrum height, and the relationship is reversed posterior to this, resembling the situation in *Gengasaurus nicosiai* Paparella, Maxwell, Cipriani, Roncacè and Caldwell 2016 [65]. For vertebra x39, the centrum height is twice as high as the neural spine height. The transition from the prezygapophysis to the pedicel is smooth, rather than abrupt. The pedicels, where visible, have a triangular shape in lateral view and are not thickened, rather they are mediolaterally narrow.

A single dorsal rib is preserved almost in entirety, probably belonging to vertebra x7, and is 81 cm long. More posterior ribs measure approximately 75 cm as preserved. Posterior to the dorsal-caudal transition, the ribs are between 2 and 5 cm in length until vertebra x60. The ribs are figure eight-shaped in cross section in the proximal portion, but this becomes less pronounced distally. This condition is typical of ophthalmosaurids, except for *Acamptonectes densus* Fischer, Maisch, Naish, Kosma, Liston, Joger, Krüger, Pérez, Tainsh and Appleby 2012, in which the ribs have a rounded cross-section with only a small groove on one side of some dorsal ribs [19] and *Mollesaurus periallus* Fernández 1999 in which the ribs bear a dorsal crest [66]. A few disarticulated gastralia fragments were preserved close to the caudal region. The fragments have a circular cross-section and are much smaller in diameter than the ribs.

#### Pectoral girdle

The scapula (Fig 8A and 8B) has a dorsoventrally expanded proximal end and a straight distal blade. The anterior third of the element widens gradually into what we interpret as a relatively small acromion process with a triangular shape in lateral view. It has a partially broken ridge dorsally on the lateral side. The acromion process is small also in *Cryopterygius kristiansenae* [25], *Platypterygius hercynicus* Kuhn 1946 [59] and *Sisteronia seeleyi* Fischer, Bardet, Guiomar and Godefroit 2014 [18]. The acromion process is taller and more prominent in *Acamptonectes densus* [19], *Sveltonectes insolitus* Fischer, Masure, Arkhangelsky and Godefroit 2011[24] and *Platypterygius australis* [26] than in *Keilhauia nui*. The shape and size of the acromion process varies in different specimens of *Ophthalmosaurus* sp. [52] (from small in e.g. OUMNH J48007 to prominent and pointed LEIUG 90986; pers. obs. LLD. Specimens assigned to *Ophthalmosaurus* because of their similarity to published specimens in e.g. [52], and that both were found in the Oxford Clay.). In anterior view, the proximal end consists of the acromion process dorsally, and ventral to it, a mediolaterally narrower section, as well as a wider coracoid facet and a more prominent glenoid contribution ventrally. Similar to *Sveltonectes insolitus* [24] and an indeterminate Albertan ophthalmosaurid specimen (TMP 92.41.01 8]) the glenoid facet does not continue on the ventral side of the scapula in *Keilhauia nui*, as it does in *Cryopterygius kristiansenae* [25].

The scapular blade has its maximum mediolateral thickness approximately midlength between the proximal and distal end. In lateral view, the distal end of the blade is angled diagonally, so that the dorsal edge reaches approximately 5 mm further distally than the ventral portion. The blade has the same dorsoventral height throughout, similar to *Acamptonectes densus* [19]; in contrast, all other ophthalmosaurids have a dorsoventrally expanded distal end. Additionally, *Platypterygius hercynicus* and *Sisteronia seeleyi* have shafts that are more thickened mediolaterally [18, 59].

The right clavicle (Fig 8C and 8D) is nearly complete and not fused to any other pectoral element. The medial margin bears a medially-projecting process along its ventral edge that is squared in anterior view. It widens dorsoventrally at approximately 15 mm from the medial edge. Anteroposteriorly the projection is thin and surrounded by a rim posteriorly. This differs from most other ophthalmosaurids, which are dorsoventrally thicker medially, often with a digitiform outline along the midline in dorsal view [6], although a morphology resembling *Keilhauia nui* has been observed in some specimens of *Ophthalmosaurus* sp. (e.g., GLAHM:V1861, pers. obs. AJR). Distal to the dorsoventral thickening, the clavicle gradually narrows again towards the distal tip. The posterior side of the clavicle is concave for articulation with the interclavicle, and this part is longer mediodistally than the distal projection, compared to *Ophthalmosaurus icenicus* [52] and *Mollesaurus periallus* [66], where the two parts are approximately equal in length. In *Janusaurus lundi* the concave section is longer than that seen in *Keilhauia nui* [29]. The distal projection is curved posteriorly, and narrows into a 10 mm wide tip, resembling the condition seen in *Ophthalmosaurus icenicus* [52], *Baptanodon natans* Marsh 1879 [67] and *Janusaurus lundi* [29]. This differs from TMP 92.41.01 (Ophthalmosauridae indet.), which is at its anteroposteriorly widest at the distal end [8].

The right coracoid (Fig 8E) is poorly preserved. In ventral view, it is reniform in outline, and as preserved approximately equal in anteroposterior length and mediolateral width. In *Cryopterygius kristiansenae* [25], the coracoid is somewhat anteroposteriorly longer than wide and is subcircular, whereas the coracoid of *Nannopterygius enthekiodon* [52] and *Sveltonectes insolitus* [24] are considerably narrower in mediolateral width than long. The anterior notch is prominent, similar to that in *Arthropterygius chrisorum* Russell 1993 [53] and *Ophthalmosaurus icenicus* [52]. The coracoid also possesses an anteromedial process, as in *Ophthalmosaurus icenicus* [52] and *Acamptonectes densus* [19]. This feature is absent in *Caypullisaurus bonapartei* [54] and in *Platypterygius australis* [26]. The glenoid and scapular facets are well demarcated, similar to *Sveltonectes insolitus* [24], but in contrast to *Acamptonectes densus*, where the two facets are not separated [19]. In *Arthropterygius chrisorum*, the angle between the two facets is less acute than in *Keilhauia nui* [53]. The scapular facet is only 45% of the length of the glenoid facet. In *Cryopterygius kristiansenae* the scapular facet is 50% of the length of the glenoid [25], and they are close to equal length in *Janusaurus lundi* [29]. The thickness of the intercoracoid facet is not possible to assess due to the preservation.

#### Forefin

The orientation of the humerus (Fig 8F and 8H) is based on McGowan and Motani [6] and Druckenmiller et al. [25]. Because of their position relative to the vertebral column, the pectoral girdle and forefin elements are interpreted to be from the right side. Of the two major processes on the humerus, we interpret the longer and more ridge-like (“plate-like”) to be the dorsal process, which makes this process more posteriorly situated than the deltopectoral crest. Following this interpretation, the postaxial edge is dorsoventrally narrower and sharper than the anterior edge in posterior view. The smallest facet is for the preaxial accessory element in most ophthalmosaurids, which also supports the interpretation of the processes. In proximal view (Fig 8H), the dorsal process is placed midway anteroposteriorly, and the more prominent deltopectoral crest is located in a relatively more anterior position. Although a small portion of the dorsal process (Fig 8F) might be lacking dorsally, the process is remarkably small in proximodistal length compared to that seen in other ophthalmosaurids, including *Ophthalmosaurus icenicus* [52] (e.g. OUMNH 48754, OUMNH 68542, LEIUG 90986 pers. obs. AJR, LLD) and *Cryopterygius kristiansenae* [25]. The dorsal process fails to meet the midpoint of the shaft, as in some specimens of *Undorosaurus gorodischensis* [58] and *Aegirosaurus leptospondylus* (SNSB-BSPG 1954 I 608, pers. obs. LLD). In *Ophthalmosaurus icenicus*, *Brachypterygius extremus* von Huene 1922 [52], *Paraophthalmosaurus saveljeviensis* Arkhangelsky 1997 [68] and *Cryopterygius kristiansenae* [25] the dorsal process extends to the humeral midpoint, and it extends further yet in *Arthropterygius chrisorum* [53], *Platypterygius americanus*, *P*. *australis* and *P*. *hercynicus* [55, 59, 63, 69]. The deltopectoral crest in *Keilhauia nui* is generally more prominent than the dorsal process (Fig 8G). As preserved, it is placed in the proximal and anterior region of the ventral surface. Similar to *Arthropterygius chrisorum* [53], *Platypterygius hercynicus* [59] and *Janusaurus lundi* [29], the deltopectoral crest of *Keilhauia nui* extends less than half of the total shaft length. In contrast, the deltopectoral crest extends distal to the mid-point in *Ophthalmosaurus icenicus* [52] and nearly reaches the distal end in *Sisteronia seeleyi* [18], *Acamptonectes densus* [19] and *Platypterygius americanus* [55].

The distal end of the humerus is anteroposteriorly wider than the proximal end. The shaft has only a weakly developed mid-shaft constriction, with the minimum width approximately 20% less than the proximal maximum width. The humerus bears three distal facets (Fig 8F and 8G). The anterior facet is partly eroded, but is interpreted to be for the preaxial accessory element, followed by those for the radius and ulna, as in the majority of ophthalmosaurids [25, 29, 30, 52, 65]. Of the three distal facets, that for the preaxial accessory element is the smallest. It is distorted, but a small rim can be seen on its anterior half. The radial facet is slightly longer anteroposteriorly than the ulnar facet and the dorsoventrally tallest of the three facets. The facet for the radius is oval in distal view, but is distorted along the anteriormost edge. The ulnar facet is posteriorly deflected, in contrast to *Gengasaurus nicosiai* [65], with an angle of approximately 120° to the radius facet. *Sveltonectes insolitus* [24], *Nannopterygius enthekiodon* [70], *Platypterygius hauthali* von Huene 1927 [69] and *Platypterygius platydactylus* [63] bear only two facets. *Maiaspondylus lindoei* Maxwell and Caldwell 2006 [71], *Aegirosaurus leptospondylus* [56], *Brachypterygius extremus* [72] and *Platypterygius americanus* [55] all have three distal facets, but the middle one belongs to the intermedium in the three first; in contrast *Platypterygius americanus* has one postaxial facet for an accessory element. *Cryopterygius kristiansenae* possesses two facets on the left humerus but three on the right [25]. A fourth facet for a postaxial element is seen in *Platypterygius hercynicus* [59] and in some specimens of *Platypterygius australis* [26]. Eight small forefin elements are associated with the humerus, of which the largest two might be the radius and ulna, based on their size and a possible articulation with the humerus.

#### Pelvic girdle

The ilium (Fig 9A–9D) is mediolaterally narrower than other ophthalmosaurids (with the possible exception of *Aegirosaurus leptospondylus*) and as both ilia are preserved and show the same feature, it is not likely that it is a taphonomic feature. The ilia are oriented similar to those in *Ophthalmosaurus icenicus* [52] with the concave side facing posteriorly. The concavity is more pronounced in *Athabascasaurus bitumineus* [22] and *Janusaurus lundi* [29]. This is probably also the case in *Aegirosaurus leptospondylus*, however the preservation is rather poor (SNSB-BSPG 1954 I 608, pers. obs. LLD). It is not possible to distinguish between the right and the left ilium. The element is equally wide anteroposteriorly and widens dorsally, making the anteroposterior length of the dorsal end 1.5 times the length of the acetabular end, which is autapomorphic among ophthalmosaurids. The end interpreted to be the acetabulum is mediolaterally thicker than the rest of the element and lacks distinct distal facets. The acetabular edge is diagonal with respect to the two parallel sides of the shaft, with the anterior side extending further ventrally.

The ischiopubis (Fig 9E) is a proximodistally short element. It is partly broken and may be slightly flattened, but the proximodistal length appears preserved in its entirety, based on the unbroken medial margin. Notably, and unique among ophthalmosaurids, the ischiopubis is slightly shorter than the femur. The ischium and pubis are completely fused without any trace of a foramen. The portion of the element that is dorsoventrally thicker than the other is interpreted to be the pubis. The complete fusion of the elements is shared with *Janusaurus lundi* [29], *Sveltonectes insolitus* [24], *Athabascasaurus bitumineus* [22], *Aegirosaurus leptospondylus* [56], *Caypullisaurus bonapartei* [17] and possibly *Platypterygius australis* [26]. This differs from *Ophthalmosaurus icenicus* [52], *Cryopterygius kristiansenae* [25], *Undorosaurus gorodischensis* [58] and *Paraophthalmosaurus* (“*Yasykovia*”) [73], all of which have a foramen or open notch (see also Discussion). The ischiopubis has an elongate trapezoidal shape, and is oriented similar to *Ophthalmosaurus icenicus* [52], *Stenopterygius quadriscissus* [6] and *Cryopterygius kristiansenae* [25], such that the anteroposteriorly widest portion is interpreted as the medial end. The medial end is dorsoventrally thicker than the acetabular end, but whether this is a real or a taphonomic artifact is difficult to determine. Similar to *Janusaurus lundi*, the medial end of the ischiopubis is straight in dorsal view [29] whereas in other ophthalmosaurids it is typically more rounded (with the possible exception of *Athabascasaurus*)[24–26, 52, 56, 58, 73] (*Caypullisaurus bonapartei*: Fernández pers. comm.). The medial edge is 1.4 times anteroposteriorly wider than the acetabular end. In contrast, in *Aegirosaurus leptospondylus* the medial end is more than twice as long anteroposteriorly than the acetabular end [56] and in *Janusaurus lundi* it is 1.8 times as long [29]. *Ophthalmosaurus icenicus* [52] and *Athabascasaurus bitumineus* [22] are also wider medially, but *Sveltonectes insolitus* is unique in having an ischiopubis equally expanded at both ends [24].

#### Hind fin

In PMO 222.655 both femora (Fig 9F–9H) are preserved; however, they are eroded and compressed (confirmed by чCT-scanning that indicates crushed trabeculae). The more complete femur is used primarily here for description, but because the processes are poorly preserved it is not possible to distinguish the left from the right. The anteroposterior orientation of the femora is based on the distinction between the tibial and fibular facets. Typically in ophthalmosaurids, the tibial facet is oriented perpendicular to the long axis of the femur, and the fibular facet is distinctly angled posterolaterally [14]. The proximal and distal ends are equally wide anteroposteriorly in dorsal view, with a modest mid-shaft constriction. The proximal end is slightly taller dorsoventrally along its anterior edge near the dorsal process. The dorsal and ventral processes are not well preserved, but do seem to have been dorsoventrally and proximodistally short compared to higher and longer processes in most ophthalmosaurids such as PMO 222.670, described below, and many specimens of *Ophthalmosaurus icenicus* ([52] and LEIUG 90986, pers. obs. LLD), as well as Cretaceous isolated femora from the Cambridge Greensand (e.g. CAMSM B58337-61 and TN1753 pers. obs. LLD and "*Platypterygius*" CAMSM B58058, pictured in [18]). However, in *Arthropterygius chrisorum*, the dorsal and ventral processes are poorly developed as in *Keilhauia nui* [53]. The femur bears two distal facets, typical of most ophthalmosaurids [24, 25, 29, 52, 53, 56, 58, 74], although *Platypterygius hercynicus*, *P*. *americanus*, *P*. *australis* [26, 55, 59] and *Paraophthalmosaurus saratoviensis* [60] have three. In *Keilhauia nui* the fibular facet faces posterolaterally, approximately 120 degrees relative to the tibial facet. Both facets have the same anteroposterior length. One of the femora was found with three associated, small rounded bones, presumably more distal elements of the hind fin, but of uncertain identity.

Ichthyosauria de Blainville 1835

Neoichthyosauria Sander 2000

Thunnosauria Motani 1999

Ophthalmosauridae Baur 1887

Ophthalmosauridae indet.

**Referred material:** PMO 222.670; posterior half of a large ichthyosaur, with a complete pelvic girdle and both femora. PMO 222.662; associated ilium, ischiopubis, femur and fibula, and articulated caudal vertebrae. PMO 227.932; a single ischiopubis, lacking only a part in the acetabular end.

#### PMO 222.670

**Fig 10:**
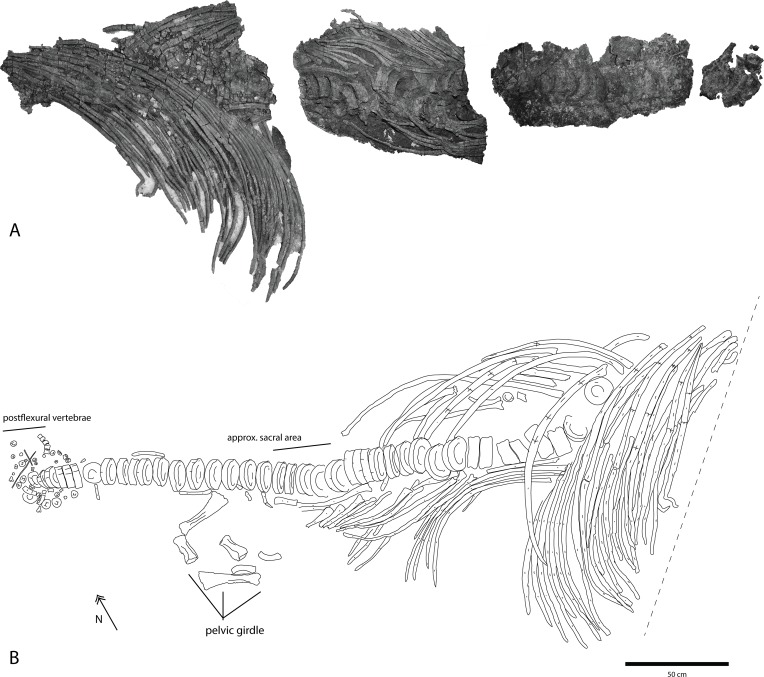
**Vertebral column of PMO 222.670** showing A, photograph of stratigraphically down side as preserved and prepared in the four jackets and B, illustration of the complete, restored, articulated series seen from the stratigraphic up side. Dashed line represents the edge of the cliff. Scale bar equals 50 cm. Modified from Delsett et al. 2016.

**Fig 11:**
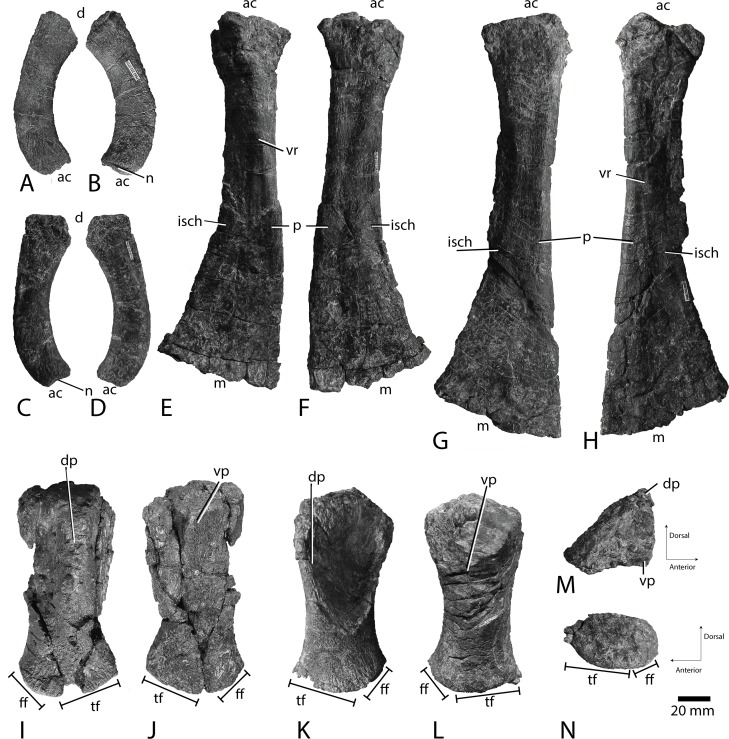
Pelvic girdle and femora of PMO 222.670. Ilium in A and B lateral (?) and medial (?) views. Posterior is to the right in A. The other ilium in C and D lateral (?) and medial (?) views. Posterior is to the right in C. Right ischiopubis in E ventral (anterior to the right) and F dorsal views. Left ischiopubis in G dorsal (anterior to the left) and H ventral views. Right femur in I dorsal (anterior to the right) and J ventral views. Left femur in K dorsal (anterior to the left), L ventral, M proximal and N distal views. Scale bar equals 20 mm. Abbreviations: **ac** acetabulum end, **d** dorsal end, **dp** dorsal process, **ff** fibular facet, **isch** ischium, **m** medial end, **n** notch in acetabular end, **p** pubis, **tf** tibial facet, **vp** ventral process, **vr** ventral ridge.

Figs 10 and 11, Table 2

**Table 2:** Selected measurements for PMO 222.670, Ophthalmosauridae indet. Lengths are given in millimetres.

**Ilium PMO 222.670/5 (Fig 11A and 11B)**	
Maximum dorsal-acetabular length (incomplete)	95
Maximum anteroposterior width, widest end	27
Maximum height (thickness), widest end	13
Maximum anteroposterior width, narrowest end	23
Maximum height (thickness), narrowest end	11
**Ilium PMO 222.670/3 (Fig 11C and 11D)**	
Maximum dorsal-acetabular length	107
Maximum anteroposterior width, dorsal end	31
Maximum mediolateral thickness, dorsal end	13
Maximum anteroposterior width, acetabular end	21
Maximum mediolateral thickness, acetabular end	12
**Ischiopubis PMO 222.670/2 (Fig 11E and 11F)**	
Maximum proximodistal length	245
Maximum anteroposterior width, medial end	85
Maximum dorsoventral thickness, medial end	11
Maximum anteroposterior width, acetabular end	54
Maximum dorsoventral thickness, acetabular end	28
Minimun anteroposterior width	33
**Ischiopubis PMO 222.670/1 (Fig 11G and 11H)**	
Maximum proximodistal length	270
Maximum anteroposterior width, medial end	88
Maximum dorsoventral thickness, medial end	9
Maximum anteroposterior width, acetabular end	57
Maximum dorsoventral thickness, acetabular end (element is compressed)	23
Minimun anteroposterior width	35
**Left femur PMO 222.670/4 (Fig 11K–11N)**	
Maximum proximodistal length	124
Maximum anteroposterior width, proximal end	68
Maximum height, proximal end	49
Maximum anteroposterior width, distal end	59
Anteroposterior length of tibial facet	16
Anteroposterior length of fibular facet	40
**Right femur PMO 222.670/6 (Fig 11I and 11J)**	
Maximum proximodistal length	126
Maximum anteroposterior width, proximal end	59
Maximum height, proximal end	54
Maximum anteroposterior width, distal end	62
Anteroposterior length of tibial facet	12
Anteroposterior length of fibular facet	49

**Locality:** Island of Spitsbergen, north side of Janusfjellet, approximately 13 km north of Longyearbyen, Svalbard, Norway. UTM WGS84 33X0519609 8695600.

**Horizon and stage:** Slottsmøya Member, Agardhfjellet Formation, Janusfjellet Subgroup, early middle Tithonian. 14.5 m above the echinoderm marker bed.

#### Preservation

PMO 222.670 represents the posterior half of an individual that landed on the seafloor on its left lateral side (Fig 10). The posterior dorsal, preflexural and some postflexural vertebrae are preserved in articulation with the dorsal ribs and associated with a complete pelvic girdle and both femora. The pelvic girdle elements and femora (Fig 11) are three-dimensional and were found close to the vertebral column in a region where the ribs experience a rapid decrease in length and then disappear (Fig 10B), suggesting that they were preserved nearly in life position. Fragments of gastralia and some severely compressed neural arches are also preserved. The preservation of the rest of the specimen is poor, due in part to compaction, the presence of siderite in the surrounding matrix and the abundant occurrence of the bivalve *Buchia* [75] on and around the skeleton. The skeleton was also covered by a thick layer of calcium sulphate [76], all of which made it difficult to assess bone texture and see facets. Finally, the posteriormost part of the skeleton, which was preserved in permafrost, is better preserved than portions of the skeleton in the active layer.

#### Total length estimate and ontogenetic status

The preserved length of PMO 222.670 is 279 cm. Based on the vertebral count of *Ophthalmosaurus icenicus* [52] and *Cryopterygius kristiansenae* [25], we estimate that 25–35 presacral vertebrae are missing, in addition to the skull. The distal end of the tail is curled up and probably not complete. Based on the length of preserved individual vertebrae (25–43 mm in anteroposterior length), the eroded presacral series had a length of 88–123 cm. Based on the body proportions of *Cryopterygius kristiansenae*, the skull was 20–25% of total body length. Assuming approximately 40 cm of the tail is missing the total body length is estimated at 510–590 cm. In *Cryopterygius kristiansenae*, which has a total length of 5.5 metres, the longest rib is 92 cm. In PMO 222.670 the longest rib exceeds 110 cm. Thus, it is likely the specimen was closer to 6, rather than 5 metres in total length. Based on its large size, the specimen is interpreted as an adult. Other ontogenetic criteria [61, 62] are not easily applicable for this specimen.

#### Vertebral column

PMO 222.670 preserves a total of 65 vertebrae (Fig 10). Sixteen centra are assumed to belong to the dorsal series and were found articulated to long dorsal ribs, 23 belong to the preflexural series and 26 to the postflexural. The floor of the neural canal is consistently about 35 mm wide at its widest point. The centra in the transition between the dorsal and preflexural series have a diameter of 80–90 mm and their anteroposterior length varies between 32–42 mm as preserved. The vertebrae are compressed to some degree, which might affect the measurements. The preflexural centra associated with the pelvic girdle have a diameter of approximately 80 mm and an anteroposterior length of 25–35 mm. Posterior to this, anteroposterior length decreases gradually.

Eighty-six dorsal ribs were preserved in articulation with the vertebrae. Many ribs are well preserved for almost their entire length. The longest ribs exceed 110 cm in length and the precaudal ribs are double-headed. Some long ribs immediately anterior to the pelvic girdle have a shallow groove on the anterior and posterior surfaces, resulting in a figure eight cross-section, whereas the remaining dorsal ribs do not show this feature. This is unusual among ophthalmosaurids, which normally have figure eight-shaped dorsal ribs. *Acamptonectes densus* and *Platypterygius americanus* also lack the figure eight shape, but *Acamptonectes densus* has a small groove on one side on some dorsal ribs and *Platypterygius americanus* and *Gengasaurus nicosiai* have dorsal ribs with spatulate distal ends [19, 55, 65]. In the few figure eight-shaped ribs in PMO 222.670, the grooves are deeper on the anterior side, and the dorsal portion more robust than the ventral portion. The distal end of all the ribs lacks the groove, as is the case for most ophthalmosaurids. Instead, the distal 25–35% of each rib is longitudinally striated. This feature has not been previously mentioned for any other ophthalmosaurid. A few disarticulated and broken gastralia, with a circular cross-section, were preserved in the caudal region.

#### Pelvic girdle

In lateral view, the ilium (Fig 11A–11D) is mediolaterally narrow and curved, presumably with the concave side facing posteriorly. The ilium is more curved than *Keilhauia nui* (PMO 222.655), but less so than *Athabascasaurus bitumineus* [22] and *Janusaurus lundi* [29]. The ilium of *Janusaurus lundi* also bears a prominent anterior process that is absent in PMO 222.670. It is not possible to interpret which element is left or right. The dorsoventral orientation of the ilium is interpreted on the basis of a small notch at one end, which is likely for articulation with the ischiopubis and is thus the acetabular end (Fig 11). The dorsal end is anteroposteriorly expanded, but less so than in *Keilhauia nui*. PMO 222.670 resembles TMP 92.41.01 (Ophthalmosauridae indet.) in that the element is anteroposteriorly widest dorsally [8]. The dorsal end is more slender mediolaterally than the rest of the element, and the ilia are in general more flattened mediolaterally than in *Ophthalmosaurus icenicus* (LEIUG 90986, pers. obs. LLD). This might be partly taphonomic, but as the two ilia are similar and other elements from the pelvic girdle are three-dimensional, it is interpreted to be an actual feature.

The ischium and pubis (Fig 11E–11H) are completely fused. The element is anteroposteriorly short, dorsoventrally thin and mediolaterally elongate. The two ischiopubes are identified as right (Fig 11E and 11F) and a left (Fig 11G and 11H). This is based on the observation that one surface is flatter than the other, which is interpreted to be the dorsal and visceral side. The dorsoventrally thicker and more rounded side is interpreted as the pubis, and is thus anterior. The left ischiopubis is longer than the right, which is probably a taphonomic artefact, as it is also less three-dimensional than the right. For the calculations, we used the mean length of each pair of elements, as was done for all other specimens which preserves both sides of a given element. In dorsal view, the anterior margin is straight, and the posterior margin is broadly concave. A prominent ridge on the ventral surface extends from the mid-point of the acetabular end towards the anterior edge at the approximate mid-length of the element. The acetabular portion is triangular in cross-section and thickened, bearing a rugose acetabular surface. The facet for the ilium is interpreted to be at the dorsoventrally tallest portion of the acetabular end. The medial end of the ischiopubis is anteroposteriorly expanded and dorsoventrally flattened. In medial view, it is slightly curved and bears a groove along its entire length, approximately 5 mm deep. The medial end is straight in dorsal view, as in *Keilhauia nui*, and 1.5 times wider anteroposteriorly than the acetabular end, similar to *Keilhauia nui*.

#### Hind fin

The femora (Fig 11I–11N) are oriented based on Maxwell et al. [14]. The left femur (Fig 11K–11N) is three-dimensional and almost complete, but slightly compressed on the dorsal side. The right femur (Fig 11I and 11J) is distorted and eroded, missing parts of the posterior margin and the ventral process, but not very flattened due to compression. The dorsal and the ventral process are approximately the same height relative to the shaft, but the dorsal process is narrower than the ventral. The processes are most similar to *Arthropterygius chrisorum* in being distinct, but not large, and approximately of the same height [53], in contrast *Sveltonectes insolitus* has relatively larger processes [24]. In *Athabascasaurus bitumineus* [22] and “*Otschevia zhuralevi”* Efimov 1998 [74] the dorsal process is more prominent, but the ventral process is larger in *Cryopterygius kristiansenae* [25]. In *Keilhauia nui* and *Platypterygius hercynicus* the dorsal process extends a third of its proximodistal length [59], in *Janusaurus lundi* [29] and *Arthropterygius chrisorum* [53], it extends midway, and in *Platypterygius americanus* it covers almost the entire length [55]. The ventral process is straight and extends approximately two-thirds of total femoral length. This morphology differs from *Caypullisaurus bonapartei*, where the ventral process extends midway [17] and less than that in *Platypterygius hercynicus* [59] and *Keilhauia nui*. The anterior margin of the femur is dorsoventrally taller than the posterior margin. The anterior and posterior margins are concave in dorsal view, the posterior more so. A large, concave area posterior to the dorsal process covers two-thirds of the dorsal surface. Where this area ends, the bone is at its narrowest anteroposteriorly. The proximal end is slightly wider anteroposteriorly than the distal. The proximal surface (Fig 11M) is triangular in medial view, with each of the three sides being roughly the same length. The outline of the distal end (Fig 11N) is oval seen in lateral view, bearing two poorly demarcated facets. The tibial facet is more than twice the length of the fibular facet. In dorsal view, the fibular facet faces posterolaterally at almost 45 degrees relative to the tibial facet, similar to *Cryopterygius kristiansenae* [25], but more than *Sveltonectes insolitus* [24] and less than *Keilhauia nui*.

#### PMO 222.662

**Locality:** Island of Spitsbergen, east side of Knorringfjellet, approximately 13 km north of Longyearbyen, Svalbard, Norway. UTM: WGS84 33X 528940 8692530.

**Horizon and stage:** Slottsmøya Member, Agardhfjellet Formation, Janusfjellet Subgroup, early middle Tithonian. 0.2 metres below the echinoderm marker bed.

Fig 12A–12F

**Fig 12:**
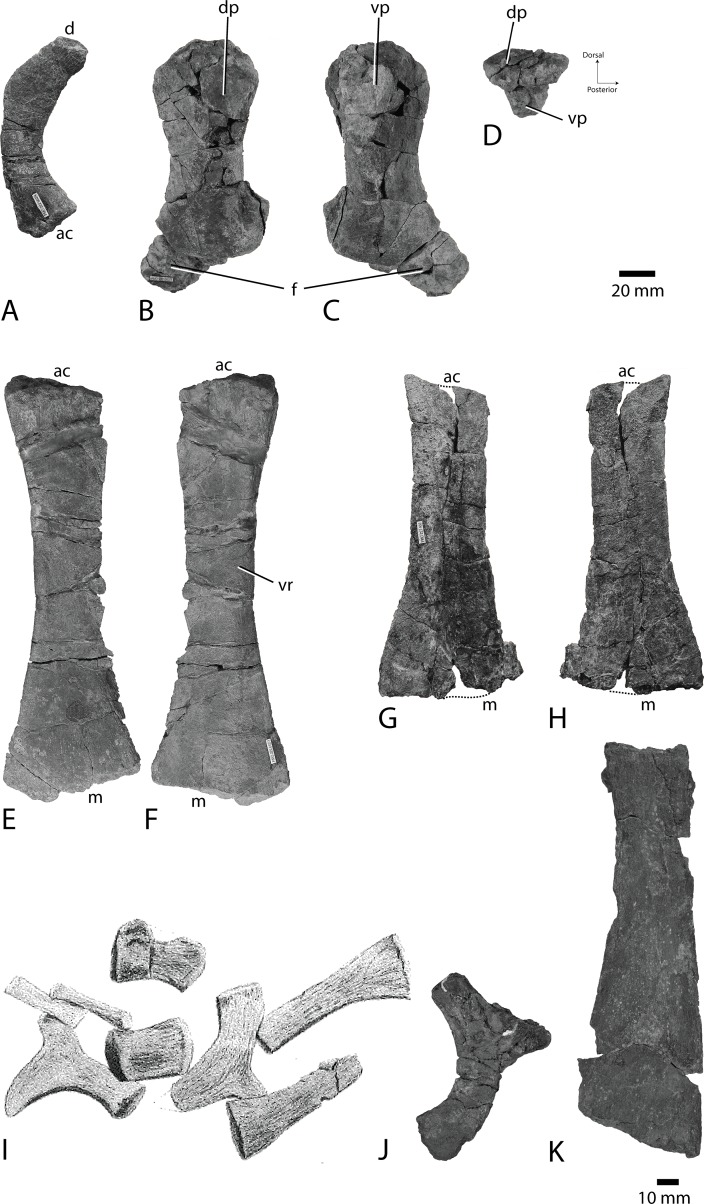
Pelvic girdles and hind fin material. Ilium of PMO 222.662 in A medial or lateral view. Posterior is to the right. Femur and fibula of PMO 222.662 in B dorsal, C ventral and D proximal views. Ischiopubis of PMO 222.662 in E dorsal and F ventral views. Ischiopubis of PMO 227.932 in G and H dorsal (?) and ventral (?) views. Dashed line shows where pieces are missing. Scale bar for A-G equals 20 mm. The Bauer (1898) specimen pelvic girdle in I, not to scale. Compare I to the right ilium of PMO 222.654 *Janusaurus lundi* holotype in J lateral view and the left ischiopubis of PMO 222.654 in K dorsal view. Scale bar for J-K equals 10 mm. Abbreviations: **ac** acetabular end, **d** dorsal end, **dp** dorsal process, **f** fibula, **m** medial end, **vp** ventral process, **vr** ventral ridge.

#### PMO 227.932

**Locality:** Island of Spitsbergen, north side of Janusfjellet, approximately 13 km north of Longyearbyen, Svalbard, Norway. UTM: WGS84 33X 0519622 8695649

**Horizon and stage:** Slottsmøya Member, Agardhfjellet Formation, Janusfjellet Subgroup, early middle Tithonian. 15.7 metres above the echinoderm marker bed.

Fig 12G and 12H

#### Ilium

From PMO 222.662, a single ilium (Fig 12A) is preserved. In lateral view, the element is curved, and the concavity is interpreted to be directed posteriorly. The curvature of the element is strongest at the approximate midpoint. Dorsal and posterior to this, the element narrows to the dorsal tip. The shape of the ilium is most similar to that of PMO 222.670, but the acetabular end is more anteroposteriorly expanded, and the posterior curvature more pronounced in PMO 222.662. The acetabular end bears a single large, posterior oval facet and a smaller rounded facet.

#### Ischiopubis

Both PMO 222.662 and PMO 227.932 preserve a single ischiopubis, which are described and compared together here (Fig 12E–12H). The ischium and pubis are completely fused and lack a foramen. PMO 222.662 (Fig 12E and 12F) is interpreted to be the left based on the criteria used for PMO 222.670 (above), but this is uncertain for PMO 227.932. PMO 222.662 is similar in overall morphology to PMO 222.670, described above. PMO 227.932 shares some features with PMO 222.670; it is anteroposteriorly and dorsoventrally slender and proximodistally elongate, and has one sharp margin and the other thickened and rounded. Based on the orientation of PMO 222.670, the thickened and rounded margin is interpreted to be located posteriorly, even though the thickened part is narrower anteroposteriorly than in PMO 222.670. Compared to the width of the medial end, the minimum anteroposterior width of PMO 227.932 is less narrow than that of PMO 222.670. The anterior margin of PMO 222.662 is sharp and slightly curved in dorsal view, and the posterior margin is dorsoventrally taller and more curved than the anterior. The posterior margin of PMO 227.932 is straight and the anterior margin curves slightly, but less than in PMO 222.670. The acetabular surface of PMO 222.662 is triangular in lateral view and is rugose. Its facets are not well demarcated and the ischiopubis is somewhat eroded in the acetabular end. A triangular area in the posteroventral area is interpreted to be the facet for the femur and the facet for the ilium more dorsally placed. The medial margin of PMO 222.662 is tallest dorsoventrally at the midpoint, and is coarsely rugose. The medial end of PMO 227.932 is expanded, but straighter than in PMO 222.670 and bears a notch along its entire distance, 5–10 mm deep.

#### Femur

A single femur is preserved in PMO 222.662 (Fig 12B–12D). The femur is constricted at midshaft and the distal end is anteroposteriorly wider than the proximal end. This differs from PMO 222.670, where the proximal end is anteroposteriorly wider, but resembles some specimens of *Ophthalmosaurus icenicus* (e.g. NHMUK 3534 and NHMUK 4531, pers. obs. AJR). It is oriented based on the processes and the facets according to Maxwell et al. [14] and the articulated hind fin of *Cryopterygius kristiansenae* [25]. The element has two processes, of which the smaller and less developed is assumed to be the dorsal process (Fig 12B). This process is barely expressed, but appears to extend past the midpoint of the shaft. The larger ventral process (Fig 12C) is more prominent and extends far past the midpoint of the shaft. Both processes are less developed than in many ophthalmosaurids, including PMO 222.670, but dorsoventrally taller than in *Keilhauia nui*. In proximal view (Fig 12D), the femoral head is an almost equilateral triangle, where the ventral process makes up one of the three corners. The distal end bears three facets, in contrast to *Keilhauia nui*, PMO 222.670 and most ophthalmosaurids, which have two facets, but similar to *Platypterygius hercynicus*, *P*. *americanus*, *P*. *australis* [26, 55, 59] and *Paraophthalmosaurus saratoviensis* [60]. One of the facets has a more acute angle relative to the proximodistal axis of the femur and is interpreted to be for the fibula. Thus, the femur is from the right side. The fibular and middle facets are longer anteroposteriorly than the anterior-most facet. The fibula (Fig 12B and 12C), which was found in articulation, is a square-shaped element.

## Results and Discussion

### Phylogenetic analysis

**Fig 13:**
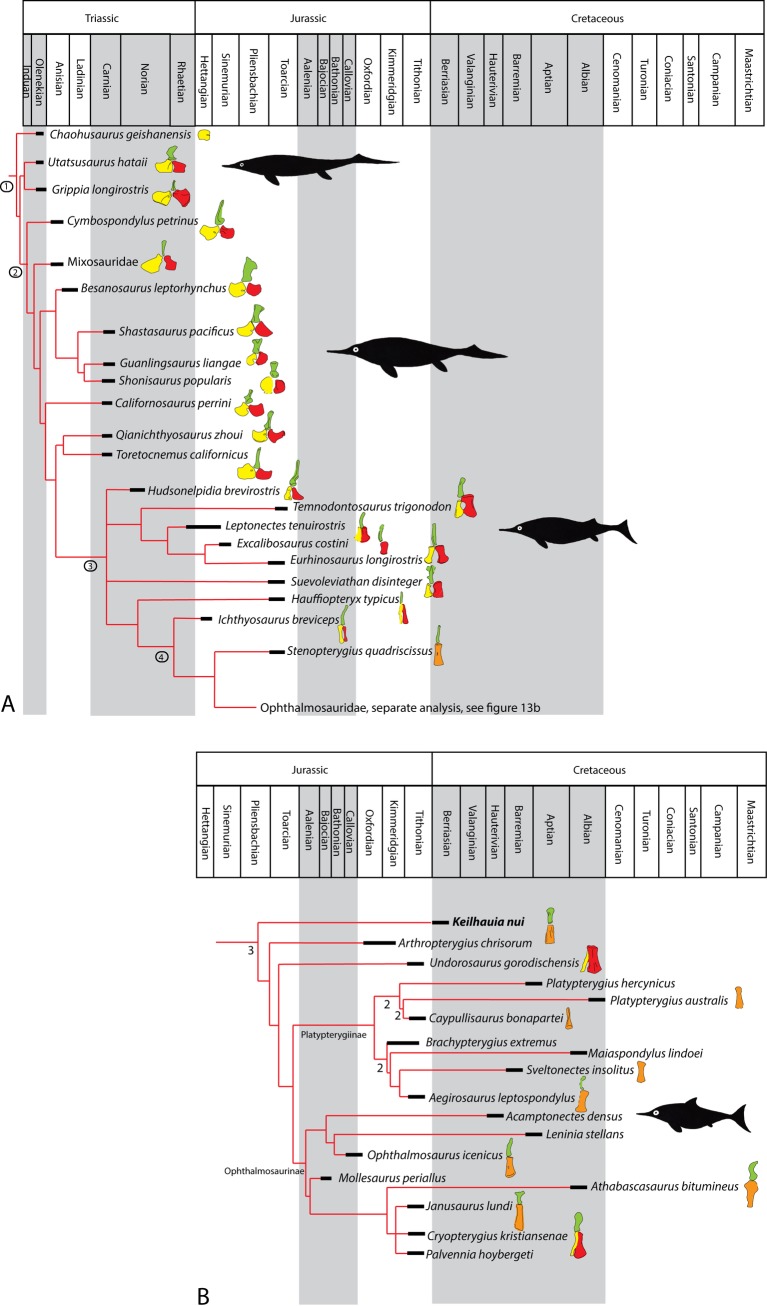
a. Time calibrated phylogeny of non-ophthalmosaurid Ichthyopterygia showing the evolution of the pelvic girdle. Articulated pelvic girdles either redrawn from the literature or based on personal observation (see references in S1 Table). Green = ilium; yellow = pubis; red = ischium; orange = fused ischiopubis. Pelvic girdles shown at different scales. Topology following Ji et al. 2016, showing only those taxa with a preserved pelvis. Clade names represented by circled numbers; 1 = Ichthyopterygia, 2 = Ichthyosauria, 3 = Parvipelvia, 4 = Thunnosauria. b. Time calibrated phylogeny of Ophthalmosauridae showing the evolution of the pelvic girdle. Articulated pelvic girdles either redrawn from the literature or based on personal observation (see references in S1 Table). Green = ilium; yellow = pubis; red = ischium; orange = fused ischiopubis. Pelvic girdles shown at different scales. Relationships following the single tree recovered in the new analysis of the Roberts et al. 2014 matrix (Length = 132; CI = 0.455, RI = 0.6.) Bremer support values for Ophthalmosauridae ≥2 are shown below the branches.

The holotype specimen of *Keilhauia nui* can be scored for 32% (18/56) of the characters in the Roberts et al. matrix [29]. The phylogenetic analysis resulted in a single MPT (Fig 13) (Length = 132; CI = 0.455, RI = 0.6). *Stenopterygius* was recovered as the sister group of Ophthalmosauridae, as in the topology presented in Ji et al. [48]. A monophyletic Ophthalmosauridae was recovered with robust support (Bremer support = 3) of which *Keilhauia nui* is the basalmost taxon. *Arthropterygius chrisorum* and *Undorosaurus gorodischensis* were the second and third most basal taxa, respectively. The remaining ophthalmosaurids are divided in two clades, Ophthalmosaurinae and Platypterygiinae, as in other analyses [29, 77, 78], although Bremer support for and within these clades is low, especially for Ophthalmosaurinae. The three described Slottsmøya Member taxa (*Janusaurus lundi*, *Cryopterygius kristiansenae* and *Palvennia hoybergeti*) form an unresolved polytomy within Ophthalmosaurinae; however, it is poorly supported (Bremer support = 1).

### Taxonomic status of new Slottsmøya Member specimens

*Keilhauia nui* can be referred to Ophthalmosauridae on the basis of a humerus (Fig 8F and 8G) possessing a facet for an anterior accessory element and the absence of notching on the anterior edge of forefin elements [6, 19]. The phylogenetic analysis also recovered *Keilhauia nui* within Ophthalmosauridae (Fig 13), but as the basalmost taxon and not nested with other Slottsmøya Member species. Given that the holotype specimen of *Keilhauia nui* (PMO 222.655) cannot be scored for any cranial characters (46% of the characters in the matrix are cranial), we interpret this topology to be suspect. Similarly, *Undorosaurus gorodischensis* was recovered as the next most basal ophthalmosaurid; it too is scored primarily on the basis of postcranial remains even though it closely resembles *Cryopterygius kristiansenae* [25, 49] in postcranial morphology, particularly with respect to its forefin.

The holotype specimen of *Keilhauia nui* (PMO 222.655) is not a juvenile form of any other known taxon from the Slottsmøya Member Lagerstätte for which there is overlapping material (*Cryopterygius kristiansenae* and *Janusaurus lundi*; *Palvennia hoybergeti* lacks comparable material). It differs from *Cryopterygius kristiansenae* in having a posteriorly deflected ulnar facet (Fig 8F and 8G) and a fused ischiopubis (Fig 9E). *Keilhauia nui* shares some features with *Janusaurus lundi*, but differs in that the humerus and femur in *Janusaurus lundi* are much more robust and possess a larger dorsal and ventral processes than in *Keilhauia nui* (Fig 9F–9H). In addition, the ilium of *Keilhauia nui* clearly lacks an anterodorsal process (Fig 9A–9D) and is instead uniquely anteroposteriorly expanded in the dorsal end.

*Keilhauia nui* has a short humerus and femur relative to total body length as well as the least developed dorsal and ventral processes of the humerus and femur compared to almost all other ophthalmosaurids [18, 19, 25, 26, 52, 55, 57, 59, 68]. The presence of two autapomorphies among ophthalmosaurids and a unique character combination in PMO 222.655 warrant the erection of a new taxon. *Keilhauia nui* is also stratigraphically much younger (Berriasian; Fig 2) than other described Svalbard ichthyosaurs and is the youngest ichthyosaur from the Slottsmøya Member described to date.

The more incomplete material, PMO 222.670, PMO 222.662 and PMO 227.932 can only be assigned to Ophthalmosauridae, due to the lack of skull and forefin material. However, the pelvic girdle and femora of PMO 222.670 (Fig 11) are different from any other described taxa, being most similar to PMO 222.662, but differing in the number of distal facets on the femur (Fig 11I–11N). PMO 222.670 preserves approximately half of the vertebral column, displaying a morphology rather typical for a large ophthalmosaurid, except the ribs that are rounded in cross-section and longitudinally striated. PMO 222.670 also shares some features with *Janusaurus lundi*, including the complete fusion of the ischiopubis (Figs 11E–11H and 12K) and femoral proportions and morphology (Fig 11I–11N)[29]. However, the ischiopubis of *Janusaurus lundi* is dorsoventrally thinner and less constricted midway than PMO 222.670 (Fig 11E–11H), and the ilium of PMO 222.670 lacks the anterodorsal process found in *Janusaurus lundi* (Fig 11A–11D).

### Relative sizes of pelvic girdle and hind fin

**Fig 14:**
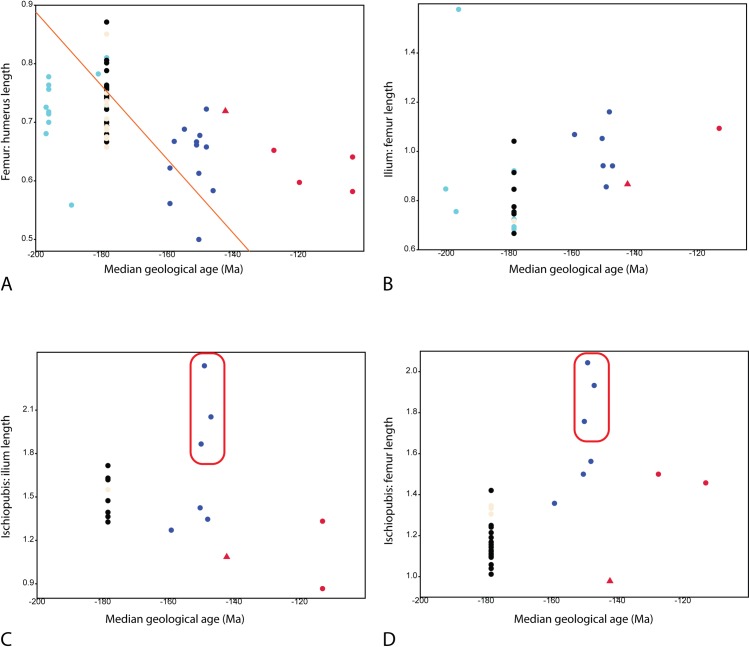
Statistical plots. *Keilhauia nu*i (PMO 222.655) represented by a triangle. Light blue = Early/Middle Jurassic specimens with a tripartite pelvis; Beige = *Stenopterygius* juvenile specimens; Black = Early/Middle Jurassic specimens with a bipartite pelvis; Dark blue = Late Jurassic specimens; Red = Cretaceous specimens. See S2 Table for specimens and references. The Slottsmøya specimens with long ischiopubis are shown in the red ellipse. A. Femur: humerus length plotted against age (Ma). There is a significant correlation (p = 1.55*10^−5^; r^2^ = 0.28 between the femur: humerus length and geological age. Orange line shows linear regression. B. Ilium: femur length plotted against age (Ma). There is no correlation (p = 0.25, r^2^ = 0.06). C. Ischiopubis: ilium length plotted against age (Ma). There is no correlation (p = 0.29, r^2^ = 0.07). D. Ischiopubis: femur length plotted against age (Ma). There is a weak correlation (p = 0.47*10^−4^, r^2^ = 0.07).

The following results summarize the relationship between the ilium, ischiopubis, femur and humerus for Jurassic and Cretaceous ichthyosaur specimens (Fig 14).

### Relative femur length

The femur length is 0.7–0.9 of humerus length in adult *Stenopterygius* (n = 30), and it is 0.5–0.7 of humerus length in Late Jurassic (n = 12) and Cretaceous (n = 5) specimens (S2 Table). A significant correlation is found (p = 1.55*10^−5^; r^2^ = 0.28) between the femur: humerus length and geological age, with the femur becoming shorter relative to the humerus through time (Fig 14A).

### Relative ilium length

No significant trend in the size of the ilium through geological time was found, when compared to either femur length (p = 0.25; r^2^ = 0.06) (Fig 14B) or ischiopubis length (Fig 4C). A specimen of *Ichthyosaurus breviceps* (CAMSMX.50187) differs from other specimens in having a long ilium relative to the femur (1.6 times as long), whereas all the other specimens have an ilium that ranges between 0.7–1.2 of femoral length. The size relationship between the ilium and ischiopubis of *Stenopterygius* is between 0.6–0.8 (n = 8; 1 juvenile) (Fig 14C).

### Relative ischiopubis length

Among ophthalmosaurids, a large range of ischiopubis length relative to ilium and femur length is found. Three Slottsmøya Member specimens (the holotype of *Janusaurus lundi* (PMO 222.654); PMO 222.670 and PMO 222.662) are unique in having very long ischiopubes; that are twice or more the length of the ilia (Fig 14C) and 1.8–2.0 times as long as the femur (Fig 14D). In contrast, *Keilhauia nui* (PMO 222.655) and the TMP 92.41.01 (Ophthalmosauridae indet.[8]) have relatively short ischiopubes (S2 Table). In TMP 92.41.01, the ischiopubis is shorter than the ilium, and in *Keilhauia nui* the ischiopubis is slightly shorter than the femur (Fig 14D).

### Evolution of the pelvic girdle in ichthyosaurs

Ichthyopterygians are believed to have become aquatic by the Early Triassic [79, 80]. However, the fossil record regarding this transition is still poorly understood, with the exception of *Sclerocormus parviceps* Jiang, Motani, Huang, Tintori, Hu, Rieppel, Fraser, Ji, Kelley, Fu and Zhang 2016 and *Cartorhynchus lenticarpus* Motani, Jiang, Chen, Tintori, Rieppel, Ji and Huang 2014 from the Spathian. The latter has been hypothesized to be able to move both on land and in water [79]. In the Triassic, early ichthyopterygians exhibited long tails and swam with axial undulation (anguilliform) [1, 5]. To obtain a high and efficient swimming speed, an organism must overcome drag, optimized by a spindle-shaped body with few irregularities, and with a ratio for length: maximum diameter of 4.5. Thus, from the Triassic to the Cretaceous, derived ichthyosaurians evolved a thunniform shape and an axial oscillatory swimming style, using a lunate tail on a narrow peduncle as the propulsive organ [1, 4, 5]. Not surprisingly, a similar length: maximum diameter value is seen in the body of extant fast-swimming dolphins and fishes [81]. Here, we attempt to focus on the evolutionary history of one important but understudied component of ichthyosaur locomotion–the pelvic girdle. In the following discussion, we provide an overview of the evolution of the three pelvic bones (Fig 13) taking into account new morphological data from several Slottsmøya Member specimens and the results of the statistical analyses (Fig 14).

#### Ilium

There are several Triassic genera with anteroposteriorly broad ilia, such as *Guanlingsaurus*, whereas other ilia were relatively straight and simple, such as *Toretocnemus* (Fig 13). *Californosaurus* bears a process on the anterior side of the ventral half of the ilium. In Jurassic parvipelvians, the ilium became narrower and simpler, with a varying degree of posterior curvature. *Suevoleviathan* is a possible exception in that it might have possessed a process (see discussion on orientation in McGowan and Motani 2003 [6]).

One important result from this study is that there is no relative size reduction of the ilium, compared to either femoral length or to ischiopubis length through the Jurassic and Cretaceous. McGowan and Motani [6: 40] noted that the ilium of *Stenopterygius* “is often relatively much smaller than it is in *Ichthyosaurus* and *Leptonectes*”, presumably in relation to the other pelvic elements. Our calculations indicate that that the ilium of *Stenopterygius* is not particularly short relative to the femur or ischiopubis when compared to other Early–Middle Jurassic taxa (Fig 14B and 14C). There is generally little variation in the ilial shape of *Stenopterygius*; the curvature is similar in all investigated specimens, and the acetabular end is somewhat mediolaterally thicker than the dorsal end.

The morphology of the ilia from the Slottsmøya Member ichthyosaurs (Figs 9A–9D, 11A–11D, 12A, 12J and 13) differ from each other and from most other known genera. *Cryopterygius kristiansenae* has an anteroposteriorly broader ilium than most other parvipelvians, whereas PMO 222.670 and PMO 222.662 have simpler, more typical ilia (Figs 11A–11D and 12A). The most unusual ilia are probably those of *Janusaurus lundi*, which have a prominent anterodorsal process (Fig 12J). They resemble a specimen figured (and later lost in WWII) by Bauer [82] as “*Ichthyosaurus posthumus*” from Solnhofen (Lower Tithonian), which is possibly closely related to *Nannopterygius* [56] (Fig 12I). The element had previously been interpreted as an ischium [56, 82], but judging from its similarity to *Janusaurus lundi*, it is likely an ilium. A specimen of *Ophthalmosaurus icenicus* (NHMUK 3013, pers. obs. AJR) and a Slottsmøya Member specimen (PMO 222.662) (Fig 12A) possess an anterior margin with a distinct dorsal bend, but lack a distinct process as seen in *Janusaurus lundi*.

#### Ischium and pubis

In Triassic ichthyopterygians, the ischium and pubis consist of two large, rounded bones (Fig 13) [83], similar to some terrestrial squamates. In most genera the obturator foramen is present. In basal ichthyopterygians, cymbospondylids as well as mixosaurids, the pubis is larger than the ischium (Fig 13). In shastasaurids, the two elements are similar in size, whereas among basal parvipelvians with a tripartite pelvis the ischium is the larger element [6, 11]. During the Early Jurassic, the ischium and pubis generally evolved into mediolaterally elongated elements. *Stenopterygius* and *Hauffiopteryx* were the stratigraphically earliest ichthyosaurs with a fused ischium and pubis, a feature also found to be subject to individual variation in *Leptonectes* and *Temnodontosaurus* [84]. In *Hauffiopteryx*, the two elements are fused only at the acetabular end, whereas *Stenopterygius* possesses only a small foramen. In *Stenopterygius*, the ischiopubis shows little intraspecific variation except in size. In *Ichthyosaurus* several species possess a pubis that is longer than the ischium, and the genus shows morphological variation in the pelvic girdle that might be important taxonomically [12].

The ischium and pubis are completely fused in the Slottsmøya Member ichthyosaurs (Figs 9E, 11E–11H, 12E–12H, 12K and 13) except for *Cryopterygius kristiansenae*, which shows a medial opening between the two elements. *Undorosaurus gorodischensis* also shows incomplete fusion [58] and an ischiopubis very similar to *Cryopterygius kristiansenae*. It has been suggested that the fusion might have been completed in life by cartilage [58]. Some specimens of *Ophthalmosaurus icenicus* exhibit a medial opening (probably between the two elements) that varies in size intraspecifically, and some specimens possess a foramen (e.g. Andrews 1910: 58; NHMUK 4754, AJR pers. obs.) [52, 85]. The loss of the ischiopubic foramen in Ophthalmosauridae occurred by the Late Jurassic, as shown in the Late Jurassic forms *Aegirosaurus leptospondylus*, *Janusaurus lundi*, *Caypullisaurus bonapartei* (Fig 13), *Paraophthalmosaurus*, PMO 222.670, PMO 222.662 and PMO 227.932.

Maisch and Matzke [11] suggested that complete proximal and distal fusion was a synapomorphy of the clade including *Stenopterygius* and Ophthalmosauridae. This is however not the result of the current phylogenetic analysis, where fusion of the ischiopubis does not appear as a synapomorphy for any clade. The varying degree of fusion of the ischium and pubis within Ophthalmosauridae could result from homoplasy or from intraspecific variation in some species. Complete fusion of the ischiopubis, including the lack of a foramen, can be observed in all known platypterygiine species for which an ischiopubis is known (Fig 13). However, complete fusion is also seen in two out of four known ophthalmosaurine species with a preserved ischiopubis, including *Athabascasaurus bitumineus* (alternatively recovered as a platypterygiine ophthalmosaurid in some studies [19]) and *Janusaurus lundi* [22, 29] and *Keilhauia nui*, whose phylogenetic position is considered uncertain. Thus, it is possible that complete fusion of the ischiopubis may be a valid synapomorphy for Platypterygiinae but not Ophthalmosaurinae [19].

The ischiopubis is long relative to the femur in Late Jurassic ophthalmosaurids, compared to *Stenopterygius*, *Hauffiopteryx* and the few known Cretaceous specimens (Fig 14D). Ischiopubes as large (twice as long or almost twice as long) compared to femur or ilium as those of *Janusaurus lundi* (PMO 222.654), PMO 222.670 and PMO 222.662 have not been described from other localities (Fig 14C and 14D). The large variation in relative size of the ischiopubis in Slottsmøya Member specimens may prove to be an informative new character in phylogenetic analyses, where the states are divided between specimens with an ischiopubis: ilium length ratio ≥ 2:1 and those with a lower ratio.

### Evolution of the pelvic girdle compared to cetaceans

In contrast to ichthyopterygians, the transition from land to water in early cetaceans is relatively well-documented in the fossil record [79]. Among cetaceans, the small pelvic girdle probably evolved because the hind fin was reduced to minimize drag as their swimming mode evolved from limb paddling to caudal oscillation [86, 87]. Early cetaceans, such as pakicetids and ambulocetids (Early- early Middle Eocene) could walk on land and swim, and had four fully fused sacral vertebrae articulating with the pelvic girdle [88]. Later protocetids (early Middle Eocene) were more aquatic, and taxa such as *Georgiacetus* retained a large pelvic girdle, but without a connection to the sacrum. In Basilosauridae, the first fully aquatic clade, the hind fin was very reduced and not connected to the vertebral column [88]. In extant cetaceans, the pelvis and hind fin is very reduced in size and is often fused into a single element [15, 16].

The evolution of the pelvic girdle and hind fin in cetaceans and ichthyopterygians exhibit a number of similarities, such as loss of contact between the pelvis and the vertebral column and the loss of the sacrum. *Utatsusaurus hataii*, a basal ichthyopterygian from the Early Triassic (Spathian), has a pelvis that articulates with two sacral ribs, but it does not seem to have been able to bear weight on land [89]. All stratigraphically younger ichthyosaur taxa lack a true sacrum [6] (but see discussion on *Grippia* in Maisch and Matzke 2000) (contra [90, 91]). Cetaceans and ichthyosaurs also reduced the relative size of the pelvic girdle, fused two or more pelvic girdle elements and lost the obturator foramen. There was clearly reduction in the relative size of the pelvic girdle and hind fin from the earliest ichthyosaurs in the Triassic to the more spindle-shaped taxa in the Jurassic, which is reflected in the clade name Parvipelvia (“small pelvis”). However, for most of the investigated length relationships of the pelvic girdle and femur there is no demonstrable trend from the earliest to the latest members of this clade (Fig 14). Although the pelvic girdle and hind fin in ichthyosaurs are not continuously reduced in size throughout their evolutionary history, or disappear entirely, these elements were nonetheless small compared to the total body size. *Keilhauia nui* (PMO 222.655) did possess a relatively small pelvic girdle, as well as a small humerus and femur, but within the range seen in parvipelvians.

### Function of pelvis and hind fin in ichthyosaurs

“Doubtless had the ichthyosaurs continued to the present time, they would have lost entirely the hind legs, as have the cetaceans.” [83: 117]. Hind fins in vertebrates have been independently lost multiple times throughout the history of life [3]. In secondarily aquatic vertebrates hind limbs have followed multiple different evolutionary pathways, including clades that retained functional limbs throughout their entire history, such as sauropterygians and mosasauroids, and others in which the hind fins have been lost or reduced, namely cetaceans and ichthyopterygians. However, an important difference between ichthyopterygians and cetaceans is that in the latter, some pelvic girdle and hind fin elements are lost completely (at least podial elements and the femur in many taxa), whereas in ichthyosaurs the complete loss of these elements has not been demonstrated [5, 7].

It is interesting to note that cetaceans completely lost their external hind fin in relatively short time span during the transition in becoming fully marine (approximately 8 million years) [88], whereas the ichthyosaurs retained an external hind fin throughout their existence in the marine realm (approximately 150 million years)[5]. That ichthyosaurs did not lose their external hind fin suggests that these elements retained a function in life, and were not only a by-product of obtaining a streamlined shape for fast swimming. Even for the small innominate of cetaceans, its retention suggests that it still had functional significance [16].

The ichthyosaur hind fin possibly had a function related to reproduction or locomotion. Male turtles and crocodilians possess a penis [92], but most fully terrestrial reptiles do not, and it is unknown in ichthyosaurs. Assuming their involvement in reproductive purposes, the hind fins could have played a role in coupling, or the pelvic girdle may have anchored the penis muscles, as in cetaceans [16]. Morphological variation in the large ischiopubes of Slottsmøya Member specimens could also result from variation in pelvic musculature. Small hind fins used in coupling has also been suggested for *Basilosaurus isis* [93]. However, the fused pelvic bones are oriented differently; anteroposteriorly in cetaceans, and mediolaterally in ichthyosaurs. It has been suggested that a flexible pelvic girdle is an adaptation to viviparity in some marine reptiles [94], and this could therefore apply to the viviparous ichthyosaurs. However, whether a flexible pelvic girdle is linked to viviparity is uncertain because most cases of viviparity in reptiles are in terrestrial species [94].

A significant correlation exists in the relationship between femur: humerus length and geological age of the specimen (Fig 14A). This confirms previous research [11], and is interesting because a larger number of late Middle Jurassic to Cretaceous specimens were included in this study. Through time, the femur becomes shorter in length relative to the humerus. This means that the relationship between the two fin pairs changed through time, possibly related to locomotion. The fins have never been the main propulsion organ in ichthyosaurs, but were probably used for steering and stabilization, with the forefin as the main steering surface [5, 7], which is most common among aquatic animals [95]. Lomax and Massare [96] suggested that the small hind fin of *Ichthyosaurus anningae* played only a minor role in maneuvering. It has also been suggested that hind fins of Late Jurassic ichthyosaurs had no function in steering or locomotion [7]. Strong correlations between serially homologous elements (among them humerus-femur) has however been seen as indirect evidence for a functional role of the hind fin similar to that of the forefin, as control surfaces [97].

## Supporting Information

S1 Text Supporting material and methods information. Supporting material and methods information, including procedure for measurements of the vertebral column, terminology used for the pelvic girdle, method for collecting morphological information about pelvic girdle elements of ichthyosaurs and cetaceans, more information on the method used to calculate the relative lengths of pelvic girdle elements, institutional abbreviations for specimens used in calculations and references for the pelvic girdle drawings in Fig 13, S1 Table and S2 Table.
(DOCX)

S2 Text Description of characters used in the phylogenetic analysis. The character list is taken from Roberts et al. 2014.
(DOCX)

S3 Text Data matrix for the phylogenetic analysis. (DOCX)

S1 Table Pelvic girdle material from ichthyopterygians Pelvic girdle material from ichthyopterygians, showing for which species either ilium, ischium, pubis or all three are described in the literature, sorted according to time period. The shape of the elements is presented in Fig 13 together with a time-calibrated phylogeny. Comments: * Taxonomic unclearity in the family, example species shown; ** Pubis not preserved; *** Undet. fragments from the pelvic girdle preserved, **** Pelvic material known for more than one species in this genus, ***** Other species in this genus might preserve pelvic girdle material. In addition, the ischiopubis and ilium are known for the Alberta specimen Ophthalmosauridae indet. TMP 92.41.01. References for the pelvic girdles mentioned in S1 Table, see S1 Text for bibliography.
(DOCX)

S2 Table Specimens used in calculations. Specimens used in calculating relative sizes of pelvic girdle elements, femora and humeri and the measurements. References given for where the measurements are taken from.
(XLSX)

S3 Table Vertebral measurements for PMO 222.655, holotype of *Keilhauia nui*. (XLSX)
