# Supplementary material for: A New Ophthalmosaurid (Ichthyosauria) from Svalbard, Norway, and Evolution of the Ichthyopterygian Pelvic Girdle
Source: PLoS One. 2017 Jan 25;12(1):e0169971. doi: 10.1371/journal.pone.0169971 (PMC5266267; doi:10.1371/journal.pone.0169971)
Supplement: S1 Text — Supporting material and methods information, including procedure for measurements of the vertebral column, terminology used for the pelvic girdle, method for collecting morphological information about pelvic girdle elements of ichthyosaurs and cetaceans, more information on the method used to calculate the relative lengths of pelvic girdle elements, institutional abbreviations for specimens used in calculations and references for the pelvic girdle drawings in Fig 13, S1 Table and S2 Table. (DOCX) [file pone.0169971.s001.docx]

**S1 Text SUPPORTING MATERIAL AND METHODS INFORMATION**

**Measurements of the vertebral column**

For the vertebral column, we use the terminology of vertebral regions defined by McGowan and Motani (1), and we also use “cervical” for the anteriormost part of the vertebral column. For PMO 222.655, measurements for width and height could not be taken while the skeleton was in articulation (except vertebra 15), and the apophyses were often not visible. Ten vertebrae (Numbers x5-x8, x13, x16-x18, x29 and x39) were removed for study and measurement, in addition to all the caudal vertebrae starting from number x44. Assignment to vertebral regions is based on Buchholtz (2) and McGowan and Motani (1). Vertebral measurements for PMO 222.655 are given in S3 Table.

The height of the neural spine in PMO 222.655 (measured along the anterior margin) and maximum anteroposterior spine length was measured when possible (dorsal neural arches, n=11 for height and n=14 for length).

Due to compressional distortion in PMO 222.670, measurements could not be taken from most vertebral centra and apophyses could only be observed on a few preflexural vertebrae.

**Terminology for the pelvic girdle**

For the pelvic girdle elements we avoid using the terms “proximal” and “distal” and instead use “dorsal” and “acetabular”. For the ischiopubis we use “acetabular” and “medial”.

**Shape of pelvic girdle elements**

For comparisons of the shape of the pelvic elements, traces were made in Adobe Illustrator for the ischium, pubis and ilium from published and/or museum-held specimens from the Triassic, Jurassic and Cretaceous (Supplementary table 1). As many genera as possible were included, but due to uncertain taxonomic status (1) the following were omitted: *Thaisaurus*, *Guizhouichthyosaurus, Callawayia* and *Mikadocephalus*. The shape of cetacean pelvic girdles were studied in the collections in the Natural History Museum in Oslo, Norway, the Natural History Museum in Bergen, Norway and from the literature. To discuss the question of how large individual variation there is in pelvic girdle element shape, specimens of adult *Stenopterygius quadriscissus* and *Ophthalmosaurus icenicus* were inspected in collections and in the literature, since these two genera are known from many specimens with preserved pelvic girdles.

**Calculation of relative lengths of elements**

Proximal-distal measurements were taken for the humerus and femur, medial-acetabular for the ischiopubis and dorsal-acetabular for the ilium. Measurements less than 150 mm were taken using calipers, and greater than 150 mm using a measuring tape. In some cases measurements were taken from the literature using high quality figures only.

Interpretations of the calculations on the relative size of the ilium should be done with some caution, since this element is often not preserved.

**Institutional abbreviations for specimens used in calculations (S2 Table)**

BRSMG, Bristol City Museum and Art Gallery, England; SNSB-BSPG, Bayerische Staatssammlung für Paläontologie und Geologie, Munich, Germany; CMN, Canadian Museum of Nature, Canada; IRSNB, Royal Belgian Institute of Natural Sciences, Brussels, Belgium; MACN, Museo Argentino de Ciencias Naturales, Buenos Aires, Argentina; MAMSPLP, Musée des Amis de la Mine in Saint-Pierre La Palud, Rhône department, France; MLP, Museo de la Plata; MOZ, Museuo Prof P. Olsacher; PMU, Evolutionsmuseet Paleontologi, Uppsala, Sverige; PRM, Regional Museum, Pugachev, Russia; SM, Schwegler Museum, Germany; SMNS, Staatliches Museum für Naturkunde Stuttgart; SRM, Saratov Regional Museum, Russia; UW, University of Wyoming, Laramie, Wyoming.

One specimen in the exhibition of BSPHGM does not have collection number, a *Stenopterygius quadriscissus* specimen. It is here named BSPHGM S.q.

**References for drawings in Figure 13, S1 Table and S2 Table**

Buchholtz EA. Swimming styles in Jurassic ichthyosaurs. Journal of Vertebrate Paleontology. 2001;21(1):61-73.

Camp CL. Large ichthyosaurs from the Upper Triassic of Nevada. Palaeontographica Abteilung A: Paläozoologie - Stratigraphie. 1980;170(4-6):139-200.

Druckenmiller PS, Maxwell EE. A new Lower Cretaceous (lower Albian) ichthyosaur genus from the Clearwater Formation, Alberta, Canada. Canadian Journal of Earth Sciences. 2010;47(8):1037-53.

Druckenmiller PS, Hurum JH, Knutsen EM, Nakrem HA. Two new ophthalmosaurids (Reptilia: Ichthyosauria) from the Agardhfjellet Formation (Upper Jurassic: Volgian/Tithonian), Svalbard, Norway. Norwegian Journal of Geology. 2012;92(2-3):311-39.

Efimov VM. Ichthyosaurs of a new genus *Yasykovia* from the Upper Jurassic Strata of European Russia. Paleontological Journal. 1999;33(1):92-100.

Efimov VM. A new family of Ichthyosaurs, the Undorosauridae fam. nov. from the Volgian Stage of the European part of Russia. Paleontological Journal. 1999;33(2):51-8.

Dal Sasso C, Pinna G. *Besanosaurus leptorhynchus* n. gen. n. sp., a new shastasaurid ichthyosaur from the Middle Triassic of Besano (Lombardy, N. Italy). Paleontologia Lombarda. 1996;4:1-21.

Fischer V, Masure E, Arkhangelsky MS, Godefroit P. A new Barremian (Early Cretaceous) ichthyosaur from Western Russia. Journal of Vertebrate Paleontology. 2011;31(5):1010-25.

Ji C, Jiang D-Y, Motani R, Hao W-C, Sun Z-Y, Cai T. A new juvenile Specimen of *Guanlingsaurus* (Ichthyosauria, Shastasauridae) from the Upper Triassic of Southwestern China. Journal of Vertebrate Paleontology. 2013;33(2):340-8.

Maisch MW. Observations on Triassic ichthyosaurs. Part VII. New data on the osteology of *Chaohusaurus geishanensis* Young & Dong, 1972 from the Lower Triassic of Anhui (China). Neues Jahrbuch für Geologie und Paläontologie Abhandlungen. 2001;219(3):305-27.

Massare J, Lomax DR. An *Ichthyosaurus breviceps* collected by Mary Anning: new information on the species. Geol Mag. 2014;151(1):21-8.McGowan C, Motani R. Ichthyopterygia. Sues H-D, editor. München: Verlag Dr. Friedrich Pfeil; 2003. 182 p.

McGowan C. A new specimen of *Excalibosaurus* from the english Lower Jurassic. Journal of Vertebrate Paleontology. 2003;23(4):950-6.

McGowan C. A remarkable small ichthyosaur from the Upper Triassic of Britich Colombia, representing a new genus and species. Canadian Journal of Earth Sciences. 1995;32:292-303.

McGowan C. A revision of the Latipinnate Ichthyosaurs of the Lower Jurassic of England (Reptilia: Ichthyosauria). Life Sciences Contributions Royal Ontario Museum. 1974(100).

Merriam JC. Triassic Ichthyosauria, with special reference to the American forms. Memoirs of the University of California. 1908;1(1):1-193.

Motani R, Minoura N, Ando T. Ichthyosaurian relationships illuminated by new primitive skeletons from Japan. Nature. 1998;393:255-7.

Nicholls EL, Wei C, Manabe M. New material of *Qianichthyosaurus* Li, 1999 (Reptilia, Ichthyosauria) from the Late Triassic of southern China, and implications for the distribution of Triassic ichthyosaurs. Journal of Vertebrate Paleontology. 2003;22(4):759-65.

Roberts AJ, Druckenmiller PS, Sætre G-P, Hurum JH. A New Upper Jurassic Ophthalmosaurid Ichthyosaur from the Slottsmøya Member, Agardhfjellet Formation of Central Spitsbergen. PLoS ONE. 2014;9(8):e103152.

Schmitz L, Sander PM, Storrs GW, Rieppel O. New Mixosauridae (Ichthyosauria) from the Middle Triassic of the Augusta Mountains (Nevada, USA) and their implications for mixosaur taxonomy. Palaeontographica Abteilung A: Paläozoologie - Stratigraphie. 2004;270(4-6):133-62.

Wiman C. Über *Grippia longirostris*. Nova Acta Regiae Societatis Scientiarum Upsaliensis. 1933;9(4):1-20.

Zammit M, Norris RM, Kear BP. The Australian Cretaceous ichthyosaur *Platypterygius australis*: a description and review of postcranial remains. Journal of Vertebrate Paleontology. 2010;30(6):1726-35.
