# Supplementary material for: A New Ophthalmosaurid (Ichthyosauria) from Svalbard, Norway, and Evolution of the Ichthyopterygian Pelvic Girdle
Source: PLoS One. 2017 Jan 25;12(1):e0169971. doi: 10.1371/journal.pone.0169971 (PMC5266267; doi:10.1371/journal.pone.0169971)
Supplement: S3 Text — (DOCX) [file pone.0169971.s003.docx]

**S3 Text** Data matrix for the phylogenetic analysis.

Format datatype=standard symbols="012345"missing=?;

Temnodontosaurus_sp. 0000000000 0000000000 0000000000 0000000000 0000000000 000000

Ichthyosaurus_communis 0000100{01}00 000011{12}100 0000000000 {01}000010000 {01}110101100 000010

Stenopterygius_quadriscissus 1001100000 0000010100 01010011?1 1100000000 10{01}0000110 ??0000

Ophthalmosaurus_icenicus 0101111101 00100101{01}1 {01}011011011 0101011101 1111110010 100010

Brachypterygius_extremus 011?0100?1 10??????12 11???1???? ?101?10010 11111100?? ??????

Maiaspondylus_lindoi ?11?1?01?1 0???????0? ??????0??? ???1?10010 1?1?0?10?? ??10??

Undorosaurus_gorodischensis 011??????? ??0??????1 ???1??0??? ?001011100 1001011000 110010

Arthropterygius_chrisorum ?????????? ????????02 11????11?1 ??01001100 ?1?1010??? 1100??

Mollesaurus_periallus 11??1?0111 ????001111 11010?0??? ?????????? ?????????? ??????

Acamptonectes_densus 11???1???? ??1?????11 11111100?1 0101{01}11101 1??1??0??? ??????

Caypullisaurus_bonapartei ????00000? 10??1?10?? ?????10??? 1111111000 11210110?? 1?0?11

Aegirosaurus_leptospondylus 000?110111 00?10001?? ?????1???? ???1?10010 11111?1021 ??0011

Platypterygius_australis 0110001001 1001012012 1101010111 0111111000 1121011020 110111

Platypterygius_hercynicus 011?100?01 ??110??1?2 ?1?10??1?1 0111111000 11210110?? 11101?

Athabascasaurus_bitumineus 10??0011?1 01100110?2 ???1110?1? ?????????? ????????20 1?????

Malawania_anachronus ?????????? ?????????? ?????????? 00??110000 ?0001?00?? ??????

Leninia_stellans ?????10?01 0010?0{01}1?1 ?0???1???? ?????????? ?????????? ??????

Sveltonectes_insolitus 101?110111 0111?1??02 11?1?100?1 110111000? ?111111021 110111

Palvennia_hoybergeti 11??00?11? ??10112??2 11??11???? ??????1??? ???1?????? ??????

Cryopterygius_kristiansenae 111?0001?0 001?1121?2 ?????1???? 0101010000 0111111000 11001?

Janusaurus_lundi 11??010?10 011?1121?2 11??11???? ??01001100 01?1110020 1100??

Keilhauia_nui ?????????? ?????????? ??????01?? 000100110? ?1??????21 00?0?0
