# Supplementary material for: A New Ophthalmosaurid (Ichthyosauria) from Svalbard, Norway, and Evolution of the Ichthyopterygian Pelvic Girdle
Source: PLoS One. 2017 Jan 25;12(1):e0169971. doi: 10.1371/journal.pone.0169971 (PMC5266267; doi:10.1371/journal.pone.0169971)
Supplement: S1 Table — The shape of the elements is presented in Fig 13 together with a time-calibrated phylogeny. Comments: * Taxonomic unclearity in the family, example species shown; ** Pubis not preserved; *** Undet. fragments from the pelvic girdle preserved, **** Pelvic material known for more than one species in this genus, ***** Other species in this genus might preserve pelvic girdle material. In addition, the ischiopubis and ilium are known for the Alberta specimen Ophthalmosauridae indet. TMP 92.41.01. References for the pelvic girdles mentioned in S1 Table, see S1 Text for bibliography. (DOCX) [file pone.0169971.s004.docx]

**S1 Table** Pelvic girdle material from ichthyopterygians

| **Period** | **Taxon** | **Ischium/pubis** | **Ilium** | **Note** | **Drawing based on** |  |
| --- | --- | --- | --- | --- | --- | --- |
| Cretaceous | *Platypterygius australis* | X | - |  | Zammit et al. 2010 |  |
|  | *Malawania anachronus* | - | - |  |  |  |
|  | *Leninia stellans* | - | - |  |  |  |
|  | *Acamptonectes densus* | - | - |  |  |  |
|  | *Sveltonectes insolitus* | X | - |  | Fischer et al. 2011 |  |
|  | *Pervushovisaurus bannovkensis* | - | - |  |  |  |
|  | *Simbirskisaurus birjukovi* | - | - |  |  |  |
|  | *Sisteronia seeleyi* | - | - |  |  |  |
|  | *Maiaspondylus lindoei* | - | - |  |  |  |
|  | *Athabascasaurus bitumineus* | X | X |  | Druckenmiller and Maxwell 2010 |  |
|  | *Muiscasaurus catheti* | - | - |  |  |  |
|  | *Keilhauia nui* | X | X |  | This study |  |
| Late Jurassic | *Arthropterygius chrisorum* | - | - |  |  |  |
|  | *Ophthalmosaurus icenicus* | X | X |  | LEIUG 90986, pers. obs. |  |
|  | *Aegirosaurus leptospondylus* | X | X |  | BSPHGM 1954 I 608, pers. obs. |  |
|  | *Cryopterygius kristiansenae* | X | X |  | Druckenmiller et al. 2012 |  |
|  | *Palvennia hoybergeti* | - | - |  |  |  |
|  | *Janusaurus lundi* | X | X |  | Roberts et al. 2014 |  |
|  | *Nannopterygius enthekiodon* | - | - | *** |  |  |
|  | *Parophthalmosaurus “Yasykovia yasykovi”* | X | - |  | Efimov 1999 |  |
|  | *Undorosaurus gorodischensis* | X | - |  | Efimov 1999 |  |
|  | *Mollesaurus periallus* | - | - |  |  |  |
|  | *Caypullisaurus bonapartei* | X | - |  | MACN-N-32, photograph from Marta Fernández |  |
|  | *Brachypterygius extremus* | - | - |  |  |  |
| Early/Mid Jurassic | *Suevoleviathan disinteger* | X | X |  | SMNS 15390, pers obs, orientation McGowan and Motani 2003 |  |
|  | *Temnodontosaurus trigonodon* | X | X | ***** | SMNS 15950, pers. obs. |  |
|  | *Leptonectes tenuirostris* | X | X | **** | CAMSM J 35279, pers. obs. |  |
|  | *Excalibosaurus costini* | X | X | ** | McGowan 2003 |  |
|  | *Eurhinosaurus longirostris* | X | X |  | Exhibited, BSPHGM, orientation Maisch and Matzke 2000 |  |
|  | *Stenopterygius quadriscissus* | X | X | **** | SMNS 15033, pers. obs. |  |
|  | *Hauffiopteryx typicus* | X | X |  | SMNS 80226, pers. obs. |  |
|  | *Ichthyosaurus breviceps* | X | X | **** | CAMSMX. 50187, pers. obs. orientation Massare and Lomax 2014 |  |
|  | *Chacaicosaurus cayi* | - | - |  |  |  |
| Triassic | *Utatsusaurus hataii* | X | X |  | Motani et al. 1998 |  |
|  | *Parvinatator wapitiensis* | - | - |  |  |  |
|  | *Xinminosaurus catactes* | - | - |  |  |  |
|  | *Grippia longirostris* | X | X |  | Wiman 1933, orientation from Maisch and Matzke 2000 |  |
|  | *Chaohusaurus geishanensis* | X | - |  | Maisch 2001 |  |
|  | *Cymbospondylus piscous* | X | X |  | Merriam 1908, orientation from Dal Sasso and Pinna 1996 |  |
|  | Mixosauridae | X | X | * | Schmitz et al 2004 |  |
|  | *Tholodus schmidi* | - | - |  |  |  |
|  | *Besanosaurus leptorhynchus* | X | X |  | Dal Sasso and Pinna 1996 |  |
|  | *Phantomosaurus neubigi* | - | - |  |  |  |
|  | *Shonisaurus popularis* | X | X |  | Camp 1980 |  |
|  | *Shastasaurus pacificus* | X | X | ***** | Merriam 1908, orientation from Dal Sasso and Pinna 1996 |  |
|  | *Toretocnemus californicus* | X | X |  | Merriam 1908 |  |
|  | *Qianichthyosaurus zhoui* | X | X |  | Nicholls et al 2003, orientation based on *Toretocnemus* |  |
|  | *Californosaurus perrini* | X | X |  | Merriam 1908, orientation from Dal Sasso and Pinna 1996 |  |
|  | *Macgowania janiceps* | - | - |  |  |  |
|  | *Himalayasaurus tibetensis* | - | - |  |  |  |
|  | *Hudsonelpidia brevirostris* | X | X |  | McGowan 1995, orientation after Maisch and Matzke 2000 |  |
|  | *Guanlingsaurus liangae* | X | X |  | Ji et al. 2013 |  |
|  |  |  |  |  |  |  |

Pelvic girdle material from ichthyopterygians, showing for which species either ilium, ischium, pubis or all three are described in the literature, sorted according to time period. The shape of the elements are presented in Fig 13 together with a time-calibrated phylogeny.

Comments: * Taxonomic unclearity in the family, example species shown; ** Pubis not preserved; *** Undet. fragments from the pelvic girdle preserved, **** Pelvic material known for more than one species in this genus, ***** Other species in this genus might preserve pelvic girdle material.

In addition, both the ischiopubis and ilium are known for the Alberta specimen Ophthalmosauridae indet. TMP 92.41.01.

References for the pelvic girdles mentioned in S1 Table, see S1 Text for bibliography.
